# Topological clustering of regulatory genes confers pathogenic tolerance to cassava brown streak virus (CBSV) in cassava

**DOI:** 10.1038/s41598-021-86806-x

**Published:** 2021-04-12

**Authors:** Thanakorn Jaemthaworn, Saowalak Kalapanulak, Treenut Saithong

**Affiliations:** 1 Bioinformatics and Systems Biology Program, School of Bioresources and Technology, School of Information Technology, King Mongkut’s University of Technology Thonburi, Bangkok, 10150 Thailand; 2 Center for Agricultural Systems Biology, Systems Biology and Bioinformatics Research Group, Pilot Plant Development and Training Institute, King Mongkut’s University of Technology Thonburi, Bangkok, 10150 Thailand

**Keywords:** Systems biology, Complexity, Regulatory networks, Reverse engineering, Robustness, Systems analysis

## Abstract

Robustness, a naïve property of biological systems, enables organisms to maintain functions during perturbation and is crucial for improving the resilience of crops to prevailing stress conditions and diseases, guaranteeing food security. Most studies of robustness in crops have focused on genetic superiority based upon individual genes, overlooking the collaborative actions of multiple responsive genes and the regulatory network topology. This research aims to uncover patterns of gene cooperation leading to organismal robustness by studying the topology of gene co-expression networks (GCNs) of both CBSV virus resistant and susceptible cassava cultivars. The resulting GCNs show higher topological clustering of cooperative genes in the resistant cultivar, suggesting that the network architecture is central to attaining robustness. Despite a reduction in the number of hub genes in the resistant cultivar following the perturbation, essential biological functions contained in the network were maintained through neighboring genes that withstood the shock. The susceptible cultivar seemingly coped by inducing more gene actions in the network but could not maintain the functions required for plant growth. These findings underscore the importance of regulatory network architecture in ensuring phenotypic robustness and deepen our understanding of transcriptional regulation.

## Introduction

The frequency of virulent disease outbreaks as a consequence of global climate change represents a major threat to crop production every year. Left unaddressed, the spread of pathogens to new areas and hosts would be catastrophic for the global staple food supply, and there exist some layers of uncertainty regarding the predictability of host–pathogen interaction. Biological robustness studies are, thus, required to comprehend the underlying disease resistance mechanisms of crops to enable robust precision breeding and guarantee food security in the future^1^. Cassava, a major staple crop that feeds over 800 million people annually^2^ faces a huge threat from the cassava brown streak virus (CBSV). In South Africa, a major production and consumption area, yield losses of up to 60 percent have been reported in susceptible cultivars^3,4^. Different degrees of success in the development of virus-resistant cultivars have been recorded, including by classical breeding, e.g. CBSV-resistant cassava cultivar Namikonga, also known as Kaleso, an interspecific backcross derivative from *M. glaziovii x M. melanobasis*^5^, and by molecular biotechnology approach, e.g. transgenic CBSV-CP hairpin-derived small RNA to immunizing resistance of CBSV and cassava mosaic virus (CMV)^6^. Pathogenic resistance is conferred by the cooperative action of responsive genes in the regulatory system, for which the pattern of cooperation is a naïve property inherited from precedents and a characteristic trait of individuals^7,8^. Nonetheless, most studies during the last decade focused on individual gene effects and proposed tolerant phenotypes based on key resistant genes.

High-throughput genome sequencing technologies have enabled us to investigate genetic inheritance in species and study cooperative adaptive responses of genes in regulatory networks via transcriptome analysis. The integration of omics data through modeling offers insights into intracellular regulatory processes beyond the readouts of measurable data at individual levels. The cooperation of responsive genes in the regulatory network is deduced from the coherent gene expression profiles and then modeled as the gene co-expression network (GCN)^9,10^. The topology of the GCN is a network characteristic that refers to the property of the regulatory system^9^. Robustness is theoretically deduced by examining if the network topology enables the system to tolerate perturbation. Two main network topological properties used to describe robustness of organismal systems are the scale-free property^11^—a “robust yet fragile” scenario where the system tolerates random perturbation^12^ but immediately collapses under targeted perturbation at a highly cooperative gene, or hub gene, and the small-world property^13^—where a high degree of cooperation within groups and closed collaboration between groups make the system promptly responsive to stimuli.

The presence of complicated and specific network topology in regulatory systems of well-adapted organisms supports the hypothesis on its requirement to enhance the capacity of organismal tolerance. Macroscopic-scale network topology, such as scale-free and small-world properties, has been used to describe robust stress-responsiveness in different organisms^8, 14,15^, while microscopic-scale topology studies, such as on clustered regulatory motifs and feed-forward/backward loop motifs, have deepened our understanding of mechanistic processes enhancing the performance of systems^16–20^. The complexity of network topology is believed to develop with the organismal evolution to improve the potential of regulatory systems. Duplication of genes with identical functions or the emergence of paralogous genes with similar or overlapping functions in evolved organisms provides flexibility for regulatory systems through the redundant functional genes^21–24^.

Amuge and team explored the mechanism of CBSV resistance in cassava based on a time-series transcriptome analysis^25^. The study identified candidate genes involved in the defense regulatory process from a set of differentially expressed genes (DEGs) during infection and proposed key candidate genes for biomarker development by comparing the DEGs of CBSV-resistant (Namikonga) and -susceptible (Albert) cassava varieties. The candidate genes offered insights into the predominant regulators but provided limited information on the underlying regulatory mechanisms against CBSV. Here, we hypothesized that resistance could not be conferred by the action of individual regulators alone but requires cooperation among the responsive genes. To evaluate this hypothesis, we analyzed the gene co-expression networks (GCNs) of resistant (R) and susceptible (S) cultivars constructed under control (C) and infected (treatment, T) conditions: GCN-RC, GCN-RT, GCN-SC, and GCN-ST, which were postulated as models of responsive gene cooperation in the specific conditions. The findings showed that cluster-based topology immensely contributed to the robustness of the GCNs, implying the importance of the gene network pattern in the robust response of regulatory systems. The network characteristic was also found to be a naïve property of the resistant variety. We propose that the distribution of hubs with a high clustering coefficient under control conditions (referred herein to demonstrate the condition of naïve plants) plays an important role in the resistance against CBSV. The increase in high-degree nodes (hubs) in response to infection could not enhance the robustness of the susceptible cultivar due to naïvely poor network connectivity (low clustering coefficient in control conditions). The proposed scenarios were supported by in silico perturbation studies of the GCN-SC and GCN-RC networks, which demonstrated the advantage of the clustered topological structure in maintaining normal regulation during perturbation. Analysis of the responses of susceptible and tolerant potato varieties to Potato Virus Y (PVY) showed similar results.

## Results

### Microscopic-scale clustered topology of the responsive gene network contributes to robustness against CBSV infection

**Figure 1:**
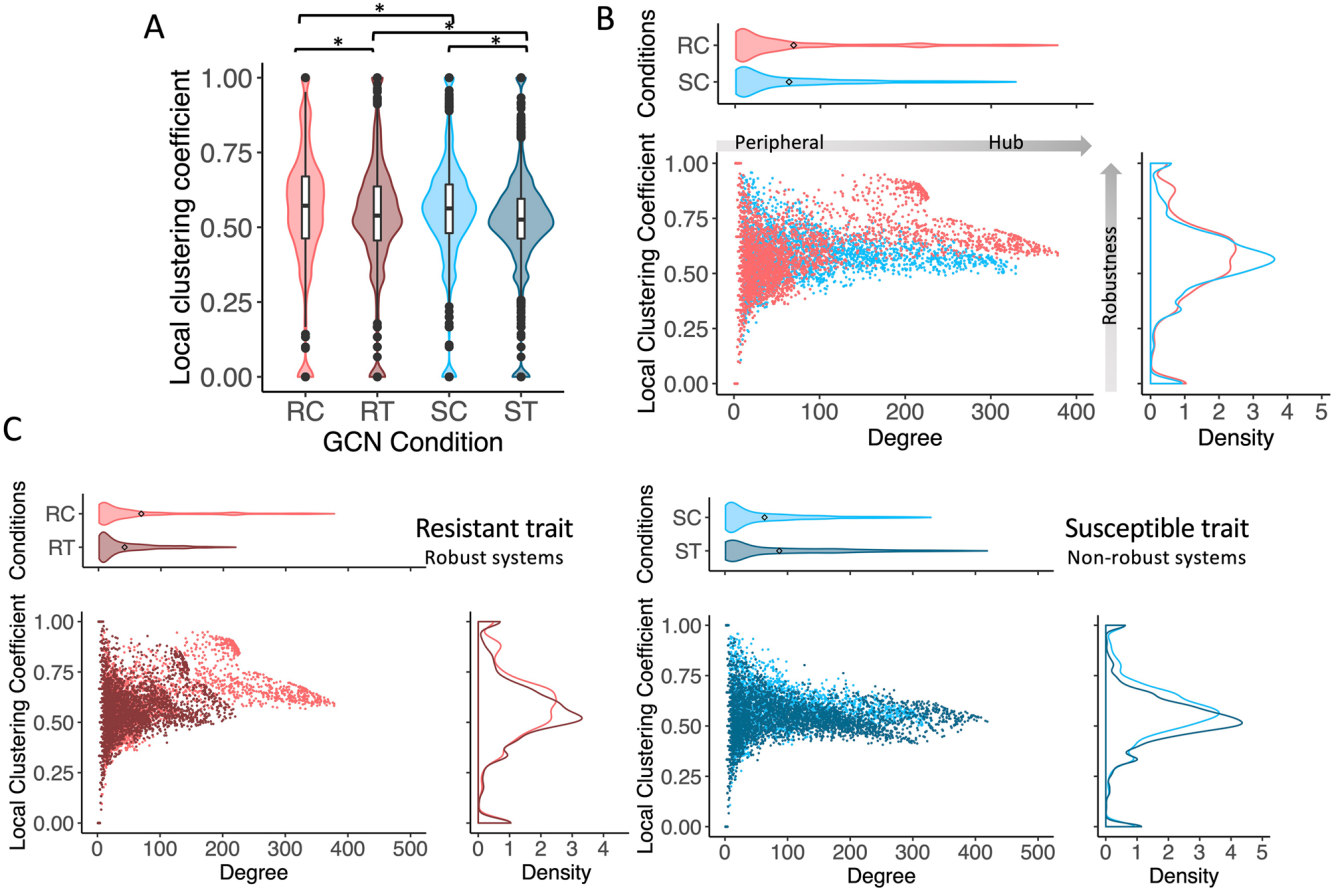
Local network properties of condition-specific GCNs; (**A**) Violin plots showing the distribution of the local clustering coefficient for all four GCNs. Asterisks (*) indicate statistically significant differences based on the Wilcoxon rank-sum one-sided test (*p*-value < 0.05). For inter-trait comparison, the median local clustering coefficients for the resistant trait were higher in both control and treatment conditions (GCN-RC > GCN-SC; GCN-RT > GCN-ST). For intra-trait comparison, the median local clustering coefficient was higher in control conditions across traits (GCN-RC > GCN-RT; GCN-SC > GCN-ST). (**B**) Scatter plot showing the distribution of local properties of nodes in GCNs under control conditions (GCN-RC and GCN-SC), and (**C**) in response to the viral infection (GCN-RT and GCN-ST). The resistant trait (C-left) responded to perturbation by dramatically decreasing the high degree nodes (hubs) and slightly decreasing the local clustering coefficient, while the susceptible trait (C-right) increased hubs and decreased the clustering coefficient in response to perturbation. The x-axis in scatter and violin plots represents the node degree, and the y-axis in scatter and density plots represents the local clustering coefficient. High-degree nodes or hub genes represent gene regulatory hubs, whereas high clustering coefficients denote highly collaborative gene association and network robustness.

The time-course of gene expression was investigated in resistant (Namikonga, denoted as R) and susceptible (Albert, denoted as S) cassava varieties infected with CBSV (treatment, denoted as T) and without (control, denoted as C). The expression profiles during CBSV infection were monitored at the beginning, early (8 days after grafting, DAG) through late (54 DAG) infection stages, 8 data points in total^25^. Differentially expressed genes across the time points were proposed as active genes functioning in response to the exposed conditions. There were 4667, 4737, 4421, and 5018 active genes identified for RC, RT, SC, and ST conditions, respectively (Additional Information 1B). The cooperative action of these CBSV-responsive genes under the study conditions was inferred from the gene co-expression networks (GCNs): GCN-RC, GCN-RT, GCN-SC and GCN-ST, and the pattern of cooperation was proposed to reflect the robustness of the system property^26, 27^. Topological analysis was performed to characterize robustness-related properties of the GCNs and test the hypothesis that robustness of the resistant variety is associated with the networking pattern of responsive genes. Results showed the macroscopic-scale topology of all networks were similar, possessing scale-free and small-world properties with comparable global clustering properties (Additional Information 1C). In contrast, when considering individual gene nodes, the distribution of the local clustering coefficient revealed topological differences at the microscopic-scale. It was found that GCNs of the resistant variety (GCN-RC and GCN-RT) contained gene nodes with higher clustering coefficients compared to the susceptible variety (GCN-SC and GCN-ST; Fig. 1A). Figure 1B demonstrates that the high clustering coefficient gene nodes in the GCN of the resistant cassava varieties (GCN-RC) tended to be highly connected to others, thus possessing high node degree value). The difference in the network topology of both cultivars under control conditions supports our hypothesis and also demonstrates that the robust pattern of cooperative gene connectivity in the network is a naïve property of the individual varieties.

By examining alterations to the gene network topology in response to the infection, it was observed that the number of genes induced was larger in the susceptible variety (GCN-ST), which showed an almost 20 percent increase in gene nodes, compared to an increase of less than one percent for GCN-RT (Additional Information 1C). The occurrence of massive genes induced by pathogenic infection was also observed in transcriptome studies of other plant species, for instance potato and apple^28, 29^. The number of differentially expressed genes were higher in more susceptible varieties, probably to alert biological processes to act against the pathogen, while targeted defenses were likely deployed by more resistant varieties.

Analysis of network dimension by edge per node ratio (*e/n*) and diameter (*d*) showed that the topology of GCNs changed inversely under infected condition, reduced diameter with more connections for the susceptible cassava variety (*d*_SC_ = 28 and *e/n*_SC_ = 30.0; *d*_ST_ = 24 and *e/n*_ST_ = 42.1) and increased diameter with less connections for the resistant cassava variety (*d*_RC_ = 23 and *e/n*_RC_ = 32.6; *d*_RT_ = 26 and *e/n*_RT_ = 19.7). Correspondingly, Fig. 1C showed an inverse change in the clustered network topology of GCNs against CBSV infection between the two varieties. GCN-RT shows a reduction in the degree of gene cooperation with a little change in the clustering topology, whereas GCN-ST shows an increase in degree of gene cooperation with a superior impact on the clustering topology (Fig. 1C and Additional Information 1C,D), suggesting an antagonistic regulatory response to CBSV infection in both varieties.

**Figure 2:**
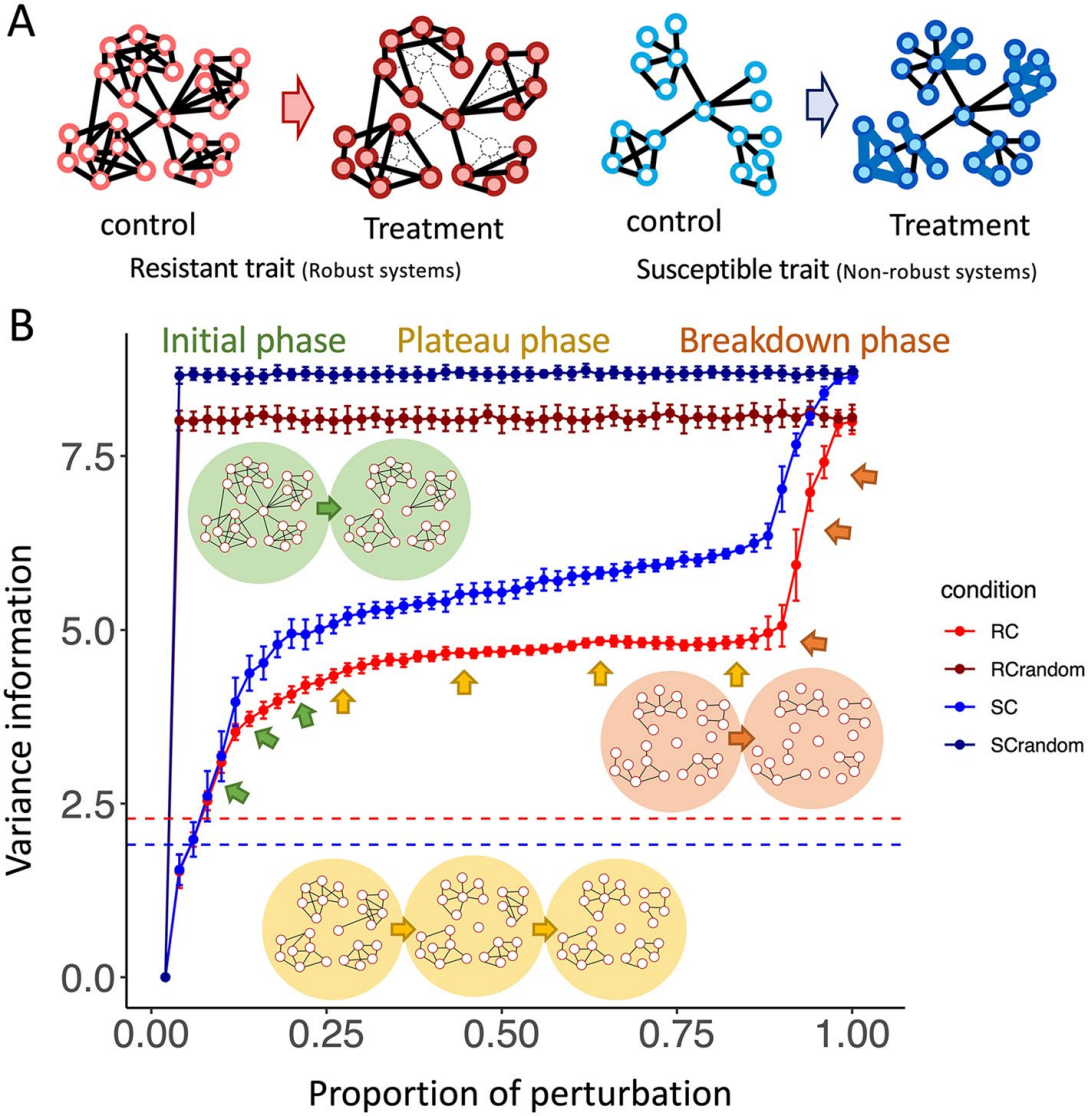
The hypothetical models of GCN network topology in resistant and susceptible trait and computational testing; (**A**) Schematic models of resistant (left) and susceptible (right) trait responses to perturbation proposed from the topological parameters investigated in each condition. Nodes represent genes in the network. Filled and unfilled nodes are genes under treatment and control conditions, respectively. Associations between genes are represented as lines; bold lines indicate associations occurring in the network, while dashed lines are associations that are missing from the previous network. (**B**) Line graph demonstrating changes in the gene community during perturbation, categorized into three different phases: *initial* (green), *plateau* (yellow) and *breakdown* phases (orange). The x-axis represents the magnitude of perturbation, which is the number of edges sequentially removed from the total number of edges. The y-axis represents the mean variance information (VI) of 30 repetitions. Error bars represent the standard deviation of 30 repetitions. Dashed lines (internal control) represent the VI calculated to reconcile the effect of the network size on the comparison. The higher the perturbation at the intercept of internal control line on VI is, the more robust the network topology.

Based on the analysis, we propose that network topology is central to the robustness of biological systems. GCNs require not only scale-free and small-world macroscopic properties but also high clustering of individual constituents. The conceptual schemes of generalized regulatory networks were proposed accordingly for resistant (Fig. 2A, left) and susceptible (Fig. 2A, right) characteristic traits. The network topology of the resistant system contains abundantly clustered motifs that are well connected, while that of the susceptible system contains clusters that are sparsely linked to each other. The hypothetical network structures are supposed to describe gene cooperative patterns of both susceptible (GCN-S) and resistant (GCN-R) varieties in response to CBSV infection. Although the infection disrupted the high degree nodes in GCN-R, leading to a decrease in the clustering coefficient, the neighboring genes were able to cooperate and maintain essential functions. The GCN-S responded to the infection by promoting the interconnection of genes (edge); nonetheless, it could not achieve robust gene cooperation as indicated by the severe symptoms observed. These hypothetical models were tested in an in silico perturbation experiment, during which the loss of gene (node) interconnections, measured in terms of mean variance information (VI), was determined for each edge removal in a sequence until complete numbers. As shown in Fig. 2B, the decomposition of the network structure can be divided into 3 phases, *initial*, *plateau* and *breakdown*. The VI rapidly increased in the *initial* phase as linkages between gene clusters were removed (marked as green in Fig. 2B). As the linkages in the network were increasingly perturbed, interconnections between genes within gene clusters were disrupted. At this stage, the overall VI of the network topology *plateaued,* since the dissociations of genes were buffered by the highly connected structure in clustered topology (marked as yellow in Fig. 2B). Finally, the VI culminated in a *breakdown* of the system when genes could no longer maintain their cooperation after almost all linkages were removed (marked as orange in Fig. 2B). The results showed that GCN-RC had relatively low VI throughout the perturbation and broke down later than GCN-SC, indicating its robustness relative to the latter; that is, its ability to better tolerate perturbation and retain cooperation of responsive genes. Furthermore, we performed motif discovery analysis to demonstrate that GCN-RC contains a higher proportion of clustered network motifs (high clustering coefficient structure) compared to GCN-SC (Additional Information 3A).

### Clustered topology of responsive genes enables resistant varieties to maintain essential functions during CBSV infection.

Analysis of network clustering was performed to determine any associations with robustness and the ability of the resistant variety to maintain essential functions and prevent regulatory function breakdown during perturbation. The modular GCNs, constructed (Additional Information 4A) using the MCODE algorithm, were examined to ascertain if the modules (representing clusters) helped reduce the disruption functions during perturbation. Under control conditions, the modular gene network of the resistant variety (RC) contained around 10 percent more number of modules (111 modules) than the susceptible variety (SC; 100 modules). The modules contained similar proportions of genes in the GCNs of both varieties, approximately 80 percent. After infection, it was observed the number and size (numbers of genes components) of modules remained unchanged for the resistant variety (RC: 111 modules with ~ 32 genes/module, and RT: 112 modules with ~ 32 genes/module), whereas the susceptible variety showed a 15 percent reduction in modules, the size of which was also larger compared to the control (SC: 100 modules with ~ 34 genes/module, and ST: 85 modules with ~ 49 genes/module). While maintaining the number of modules and gene components, the number of gene associations within each module of RT was reduced by 20 percent (module density: RC = 4.13 and RT = 3.34). For the susceptible variety, the module size differed with an increase in the average density of the network (module density: SC = 3.38 and ST = 3.69). The analysis showed that the resistant variety minimized the perturbation effect by restructuring intra-modular linkages to keep the global network structure, in contrast to the non-buffering response of the susceptible variety in altering the module number. Results of the structural analysis suggest the clustered topology helped attenuate the effect of infection and enhanced tolerance by maintaining normal cellular regulation.

**Figure 3:**
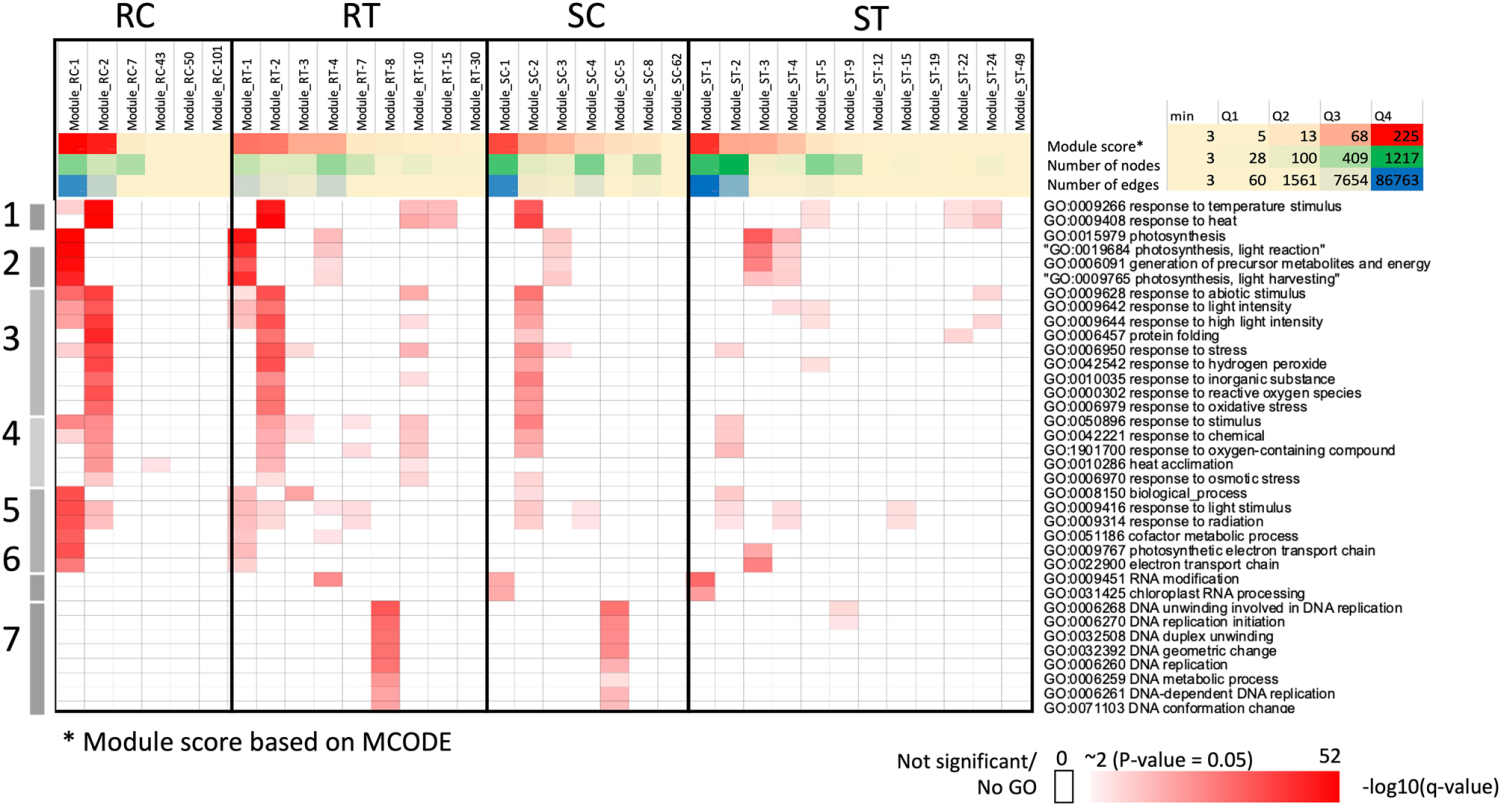
Functional analysis of modular gene networks. Heatmap demonstrating highly enriched biological process functions of each module (–log_10_Q-value more than 10; see Additional Information 5A for full analysis results). The columns represent modules, rows represent functions determined for the seven clades: clade1 for heat response, clade2 for photosynthesis, clade3 for stress response, clade4 for the chemical response, clade5 for the light response, clade6 for RNA processing and clade7 for DNA processing, and colors represent the significance (–log_10_Q-values). Modules RC-1, RC-2, RT-2, RT-10, SC-2, ST-5, ST-22, and ST-24 were identified as heat response and stress response modules (both clade1 and clade3). The major heat response modules for each condition, namely Module_RC-2, Module_RT-2, Module_SC-2, and Module_ST-5, were determined by the largest module size (number of nodes in the module).

Next, we hypothesized that modules in the modular gene networks hold specific functions that are essential for normal growth and development in plants during infection. For each module, enriched GO terms of constituent genes were analyzed, and module functions were inferred. Figure 3 shows unsupervised clustering of enriched functions in functional modules of RC, RT, SC, and ST networks (see entire results in Additional Information 5A). The results showed that the functions of modular genes were similar across conditions, indicating their importance in plants. These functions were related to photosynthesis (e.g. photosynthetic light reaction/harvesting; Module RC-1, Module RT-1, Module SC-3, and Module ST-3) and stress responses (e.g. responses to abiotic stimuli, reactive oxygen species, and chemicals; Module RC-2, Module RT-2, Module SC-2, and Module ST-5). As shown in Fig. 3, the resistant variety was able to retain these basic functions better than the susceptible variety.

Correspondingly to the study by Amuge et al.^25^, our results highlighted the primary role of heat shock proteins (HSPs) in CBSV resistance. Figure 3 shows that while the susceptible variety could not well maintain the function “response to heat and temperature” that was related to HSPs, the resistant variety fared better in comparison (module RC-2, RT-2 and SC-2). The HSP system is crucial for protein folding or protein modification, an important mechanism of resistance against CBSV infection^25^. Moreover, more numbers of HSP genes were found in GCNs of the resistance variety (Additional Information 5A (B)), corroborating Amuge et al.^25^, who reported larger numbers of differentially expressed HSP genes in a resistant variety infected with CBSV. It was also found that HSP genes in GCNs had explicitly high local cluster coefficients comparing to others in the network, suggesting their significance for topology-based robustness of the resistant system (Additional information 5A(C)).

Further investigation of the gene expression profiles revealed a failure of modular regulatory functions in ST, while these were maintained in RT (Additional Information 6A). In addition, the motif discovery analysis of modules in RC and SC (Additional Information 7A) showed that the resistant variety could keep its regulatory functions through the clustered network topology. In the resistant network, we also found direct cooperation between the response module and pathogenic recognition module, which consisted of NBS-LRR genes reported by Amuge et al.^25^. It may imply an early response behavior of resistance after infection. This direct cooperation was not observed in the susceptible network under control conditions, which corresponds to a late response behavior as demonstrated in Additional Information 8A. These findings could explain the distinct responses of both varieties against CBSV.

**Figure 4:**
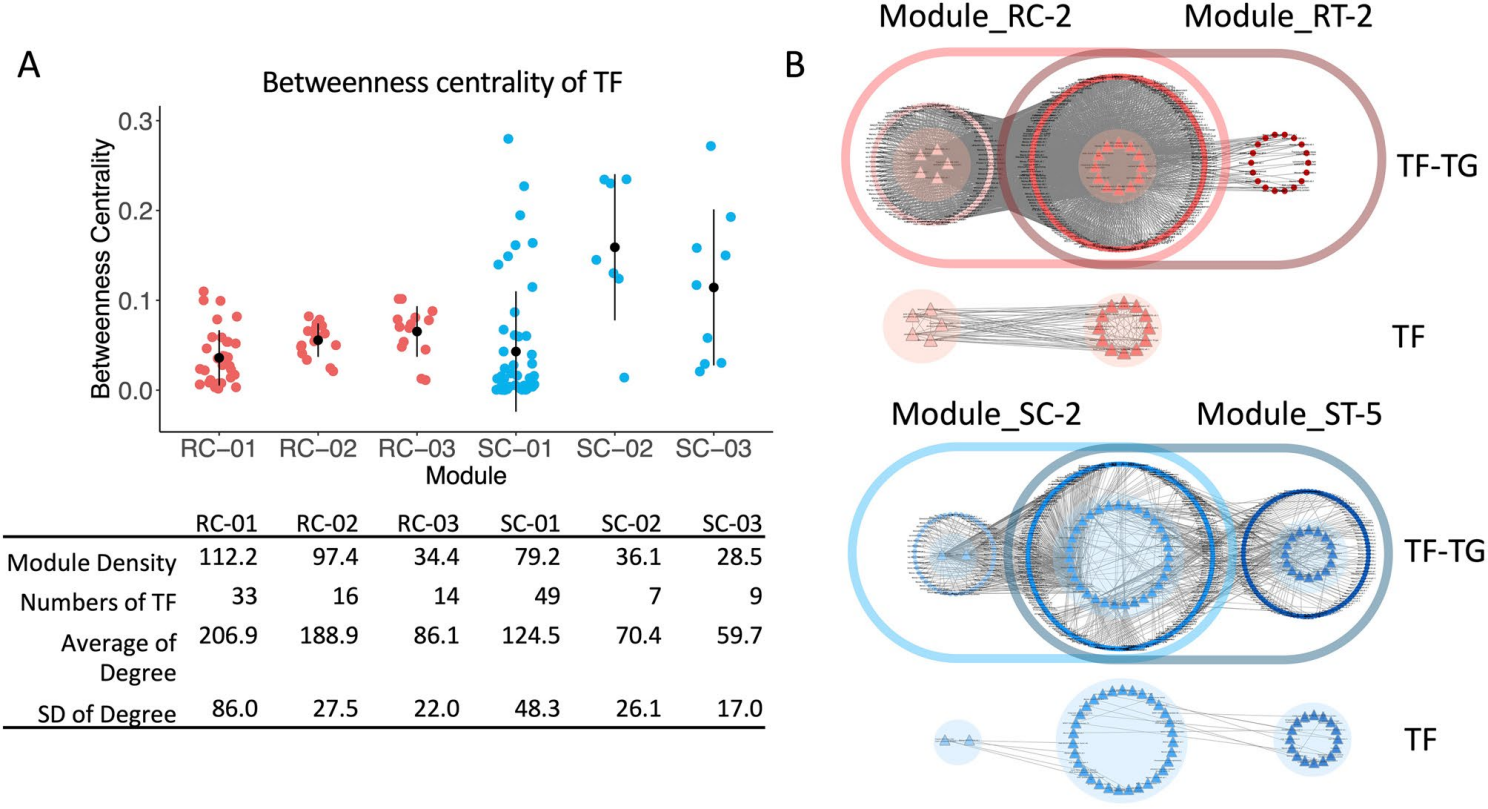
Betweenness centrality of TFs in TRN modules. (**A**) Jitter plot demonstrating the betweenness centrality of transcription factors (TFs) in the TRN modules. The standard deviation (SD) of the betweenness centrality of TFs in the resistant TRN was lower than for the susceptible TRN under control conditions. This suggests that other TFs in the resistant modules can alternatively be used for regulating target genes (TG) when the preferred TFs are perturbed, while the susceptible modules require specific TFs for regulating the TGs. (**B**) Venn diagram demonstrating TRN modules for each trait under control and infection conditions. Triangular nodes represent TFs, and circular nodes represent TGs. Only TF-TF associations are shown below the Venn diagram. Alternative TFs can be used during system perturbation to regulate genes via the complex topology of the resistant TRN in the naïve state. The susceptible TRN requires specific transcriptional regulation and has to activate new TFs to respond to infection.

Considering the contribution of clustered topology to biological transcriptional regulation, the transcriptional regulatory networks (TRNs) of top 10 modules with high MCODE scores were reconstructed, with the inclusion of associations of transcription factors (TFs) and target genes (TGs) (Additional Information 9A). We hypothesized that the topology of TGs and TFs associations might be related to the rigorous regulation of TG transcription by TFs, which leads to robustness to stress stimuli. Here, the betweenness centrality of TFs in TRN modules was examined to determine the pattern of transcriptional regulation in the cassava varieties. The results showed a slight variation in the betweenness centrality of TFs in the TRN modules of the resistant variety, with a high node degree on average, in comparison to the sizeable variation found in the susceptible variety (Fig. 4A and Additional Information 9A). It is suggested that the resistant variety has more rigorous regulatory systems. Transcription of some TGs was regulated by multiple TFs, providing flexibility and redundancy into the regulatory regime, both of which are important for a robust system^30^. Moreover, the tight distribution of the betweenness centrality may imply a closed regulation of the system from the influence of TFs in modules where the TF-TG association is cross-connected. In such systems, the expression of one TG is typically influenced by various TFs, and each TF controls the transcription of many TGs. This enables synchronized transcriptional regulation in plants for a prompt response to infection. As demonstrated by the analysis of TRNs modules with similar enriched functions (Fig. 4B), the TFs in the TRN module for the resistant variety under infection (Module_RT-2) were all in action under control conditions (Module_RC-2), while just a few active TFs in TRN module for the susceptible variety (Module_ST-5) were in action under control conditions (Module_SC-5). These findings further indicate that the robust system derived by complex regulation is a naïve property of plants.

**Figure 5:**
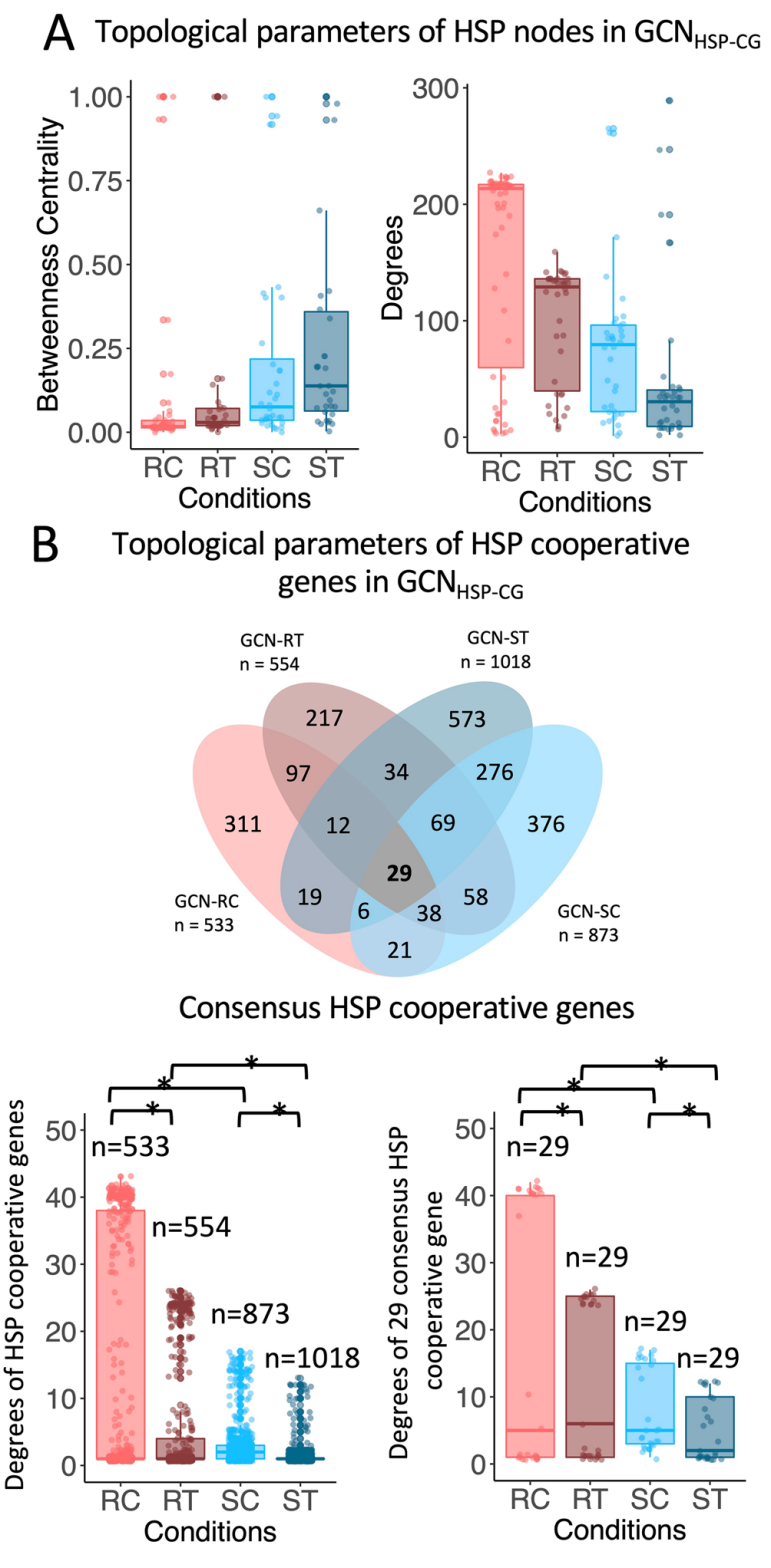
Topological analysis of HSP gene co-expression networks. (**A**) Boxplot showing the distribution of topological parameters, betweenness centrality and node degree distribution, of HSP genes in GCN_HSP-CG_. GCN-RC had the lowest betweenness centrality and highest node degree, indicating that HSPs are highly cooperative and can alternatively be used for defense purposes. (**B**) Boxplot showing node degree distribution of HSP-cooperative genes: (B-top) HSP-cooperative genes in the four conditions, (B-bottom-left) node degree distribution of HSP-cooperative genes in GCN_HSP-CG_ (n_RC_ = 533 genes, n_RT_ = 554 genes, n_SC_ = 873 genes, n_ST_ = 1018 genes), (B-bottom-right) node degree distribution of HSP cooperative genes present in all four conditions of GCN_HSP-CG_ (n = 29 genes). Asterisks (*) indicate statistical differences based on the Wilcoxon rank-sum one-tailed test (*p*-value < 0.05). It was evident that GCN-RC > GCN-RT, GCN-SC > GCN-ST, GCN-RC > GCN-SC and GCN-RT > GCN-ST.

Furthermore, we tested the proposed scenarios with the well-known regulatory sub-system of heat shock protein-encoding genes (HSP) in response to infection. In the original study by Amuge et al.^25^, HSP genes were proposed to be important in the defense against CBSV. The GCNs of HSP genes (GCN_HSP_) and their cooperative genes (GCN_HPS-CG_) were constructed for all studied conditions (Additional Information 10A). Topological analysis of GCN_HSP_ and GCN_HSP-CG_ showed that the cooperative regulation of these genes was more elaborate in the resistant variety due to the higher clustering pattern, and the regulatory network structure remained unchanged under infection, suggesting a more robust defensive system. The cross connection between the clustered topology of the gene network was also well demonstrated by the betweenness centrality and node degree of HSP and the HSP-cooperative genes (HSP genes and their first neighbor genes) in GCN_HSP-CG_. Figure 5A exhibits similar trends to those observed in the TRNs shown in Fig. 4A. Cooperation of HSP genes in GCN_HSP-CG_ of the resistant variety showed a low distribution of betweenness centrality and a high degree of connection (Fig. 5A), while HSP-cooperative genes had a high node degree, indicating a highly cooperative manner of regulation (Fig. 5B). It is thus presumed that the topology of GCN_HSP-CG_ enhanced the tolerance of the resistant variety to CBSV infection through the redundant regulatory mechanisms.

### Microscopic-scale clustered topology of responsive gene networks of Potato Virus Y resistant cultivar

**Figure 6:**
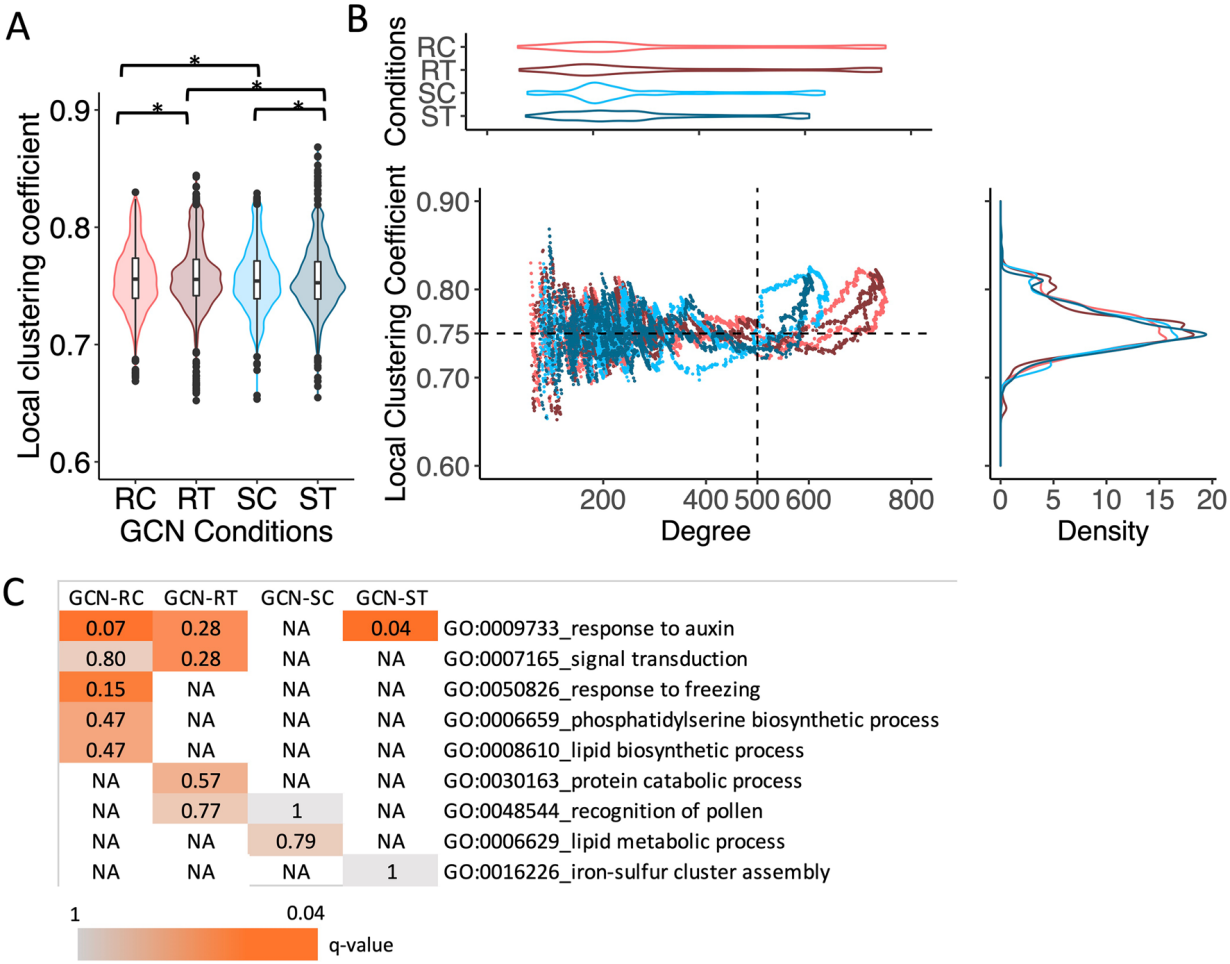
Demonstration of the hypothetical robustness based upon the cluster topology of Potato Virus Y (PVY) resistant and susceptible cultivars. (**A**) Violin plots showing the local clustering coefficient distribution of resistant and susceptible potato cultivars infected with PVY. Asterisks (*) indicate statistically significant differences based on the Wilcoxon rank-sum one-tailed test (*p*-value < 0.05). The median local clustering coefficient of GCN-RC > GCN-RT, GCN-SC > GCN-ST, GCN-RC > GCN-SC and GCN-RT > GCN-ST. (**B**) Scatter plots showing the distribution of local properties of all nodes in the naïve networks; the x-axis in the scatter plot and violin plot represents the node degree, and the y-axis in the scatter plot and density plot represents the local clustering coefficient. (**C**) Heatmap showing the significance of GO enrichment (Q-value) of predominant genes in GCNs with high-degree nodes (degree > 500) and high clustering coefficients (c > 0.75).

A similar analysis was performed using Potato Virus Y (PVY) resistant (Premier Russet) and susceptible (Russet Burbank) potato cultivars to demonstrate that clustered topology of the GCNs is associated with the adaptive responses of plants to infections. The GCNs were constructed from time-series transcriptome data of the two potato varieties under control and PVY conditions. The resistant potato variety showed a higher local clustering coefficient, implying more cooperative regulation of genes in GCN-RC than in GCN-SC (Fig. 6A and Additional Information 11A). Figure 6B demonstrates alterations to the GCN topology in response to PVY. There was no obvious difference in the responses of the resistant (GCN-RC and GCN-RT) and susceptible (GCN-SC and GCN-ST) varieties in this case, probably due to the limited number of data points in the transcriptome data (3 time-points), when compared to of the cassava data (8 time-points). However, functional analysis of predominant genes in the GCNs, based on the high local clustering coefficient and node degree, showed a distinction in the enriched functions relevant to defensive regulation, i.e. the response to auxin by the resistant variety (Fig. 6C). This corresponds to the study by Goyer et al.^29^, who proposed the importance of auxin response in the defense mechanism against PVY in potato. Moreover, crosstalk of hormonal signals between auxin, salicylic acid, and ethylene is known to be important for plant pathogen defense^31^. Our results showed that networks of the resistant potato variety for both control and infection conditions (GCN-RC and GCN-RT) contained signal transduction functions required for complex regulations and stress resistance, but not the susceptible variety. In addition, the potato GCNs were applied to construct condition-specific transcriptional regulatory networks (TRNs) to further support our hypothesis. The results showed that local clustering coefficients of TFs regulation (TRN_TF-TF_) for RC and RT were higher than for SC and ST (Additional Information 11B–E), and are comparable with the GCN analysis for potato (Fig. 6) and cassava (Fig. 1).

## Discussion

Gene association analysis is widely used to envision how genes work cooperatively in regulatory systems under prevailing conditions^9, 10^. It has enabled insights into plant regulatory responses to stress, for example the responses of Arabidopsis^32^ and apple^28^ to bacterial infection and cotton^33^ and wheat^34^ to drought stress. Gene co-expression networks facilitate the holistic inference of associations based upon concurrence between gene expression patterns. Although the analysis may neither indicate any physical interaction, e.g. direct binding of regulatory genes, nor actual co-regulation, yet the resulting network often enables subsequent identifications^35^. In a co-expression analysis of starch metabolism in Arabidopsis under diurnal conditions, Ingkasuwan et al.^35^ identified indeterminate domain 5 (AtIDD5: At2g02070) and constans-like (COL: At2g21320) as TFs co-regulating starch synthase 4 (SS4) expression, which was validated by quantitative expression analysis in mutant lines. Also, co-regulation of the KNOX1 TF and phytohormone-related genes was proposed as active during cassava storage root initiation, which was then validated by exogenous treatment of phytohormone and qRT-PCR expression analysis^36^.

The topology of a gene association network, e.g. gene co-expression network, represents a characteristic of the system. Network topology is a theoretical concept widely used to dissect the property of regulatory systems and gain more insights into the phenotypes of organisms^19, 20, 37, 38^. Marais and team^38^ linked the regulatory roles of stress responsive genes in plants to the network topology. They found that drought stress responsive genes were highly associated with others and were often located at the center of the GCN, while cold stress responsive genes were less associated and located at the periphery of the network. Moreover, studies have shown that the metabolic network is a well-designed topology-based system. The topology of the metabolic network is believed to enhance metabolic robustness, as recently proven by Kawakami et al.^39^ in a study on the topological analysis of a genome-scale metabolic model of yeast in response to 26 stimuli.

In this study, network topology analysis was introduced to unravel the different canonical regulations in the regulatory systems of CBSV-tolerant and CBSV-susceptible cassava varieties. Gene co-expression networks were constructed for individual varieties to represent the cooperative network of responsive genes under control and infection conditions. The topology of the GCNs (GCN-RC, GCN-RT, GCN-SC and GCN-ST) was characterized and contrasted under control and infection conditions to determine if it contributed to the robustness of the system in response to the pathogen. The results indicated that the densely cooperative genes, inferred from the local clustering coefficient, in the GCN of the tolerant variety might have contributed to its robustness against the infection (Fig. 1, Additional Information 1C). The generalized network structures were proposed for distinctly robust systems (Fig. 2A) based on the observed topology of their GCNs (Fig. 1). The hypothetical models were rigorously examined by in silico perturbation (Fig. 2B) and network motif discovery (Additional Information 3A), which corroborated the association of topological clustering with the robustness of systems.

The highly clustered gene network topology was hypothesized to act as a buffer to help attenuate function disruption during perturbation. The hypothesis was tested by functional analysis of the modular gene co-expression networks. The results showed that the highly clustered module structure of the resistant cassava variety helped retain essential cellular functions, such as photosynthesis and responses to light and stress during infection, which were severely disrupted in the susceptible variety (Fig. 3). The modular gene co-expression networks suggest a linkage between immune response against the CBSV infection, light response and photosynthetic processes in cassava, though they are not relevant pathways. The connection of photosynthesis and light conditions to biotic responses in plants, mostly through complex hormone signaling and energy molecule supply^40–42^, has been reported in various studies. Photosynthesis generates carbon substrates and energy molecules (ATP and NADPH) and supplies these resources to synthesize important primary metabolites, antimicrobial compounds, and defense-related hormones such as abscisic acid, ethylene, jasmonic acid, and salicylic acid^42^. In infected plants, the accumulated defense-related hormones such as ABA^43^, JA^44^ and NO^45^ in turn influence photosynthesis. Induction of bacterial defense to *Pseudomonas syringae* in Arabidopsis was reported to be a light-dependent process, suggesting that plants response to diseases may vary depending on the time of infection. The study also showed that pathogenesis-related genes involved in defense mechanisms might be linked by the phytohormone salicylic acid^46^.

The hypothesis that a clustered network pattern is required for a robust response was also tested with TRNs of the two cassava varieties during CBSV infection. Topological analysis of the networks showed that the TRN of the resistant variety had tight TF-TG cooperation. One target gene was regulated by multiple TFs (Fig. 4). Redundancy enhances flexibility, enabling the system to tolerate perturbations^30, 47–49^. Such a regulatory response involving redundant transcriptional regulators and gene functions is typically observed in plant species^49–51^ and allows plants to handle perturbations better. In Arabidopsis, redundant regulation of development is demonstrated by the partially overlapping functions of TEOSINTE-LIKE1, CYCLOIDEA, and PROLIFERATING CELL FACTOR1 (TCP) transcription factors in regulating leaf senescence^47^, and BRASSINAZOLE-RESISTANT(BZR) and BES/BZR HOMOLOG (BEH) transcription factors in controlling embryonic stem development^49^.

Corresponding to transcriptome analysis of Amuge and team^25^, we found that genes related to infection recognition (LRR-proteins, chaperon and HSPs), phosphorylation and transcriptional regulation (TF genes) were important in response to CBSV infection. Our analysis showed that these genes were not just individually relevant, but also their contribution to the topology of the responsive gene network was crucial. We showed that LRR proteins and the chaperon/HSP-enriched module were linked in the gene cooperative network of the resistant variety, while this linkage was absent in the network of the susceptible variety during infection (Additional Information 5A(A) and 8A). The importance of HSPs for the topology of the robust cooperative network was highlighted by the high local clustering coefficient compared to other responsive genes. Considering GCN_HSP_ and GCN_HSP-CG_, the average clustering coefficient of the resistant variety’s networks was high and slightly declined under infection, unlike a drastic decrease in the susceptible variety’s (Additional Information 5A(B) and 10A). As supporting evidence, other studies have reported that HSP and small chaperon genes are involved in multi-stress resistance^52–54^.

The contribution of the clustered cooperative gene pattern to biological robustness was validated with the GCNs of potato infected with PVY, in order to assure the conclusion acquired from the analysis of the two cassava cultivars (one susceptible and one resistant varieties). The GCN-RC showed a higher local clustering coefficient than GCN-SC (Fig. 6A and Additional Information 11A), corresponding to the findings on cassava resistance and susceptibility to CBSV. However, changes in the topology of the GCNs after infection were not obviously different between the resistant (GCN-RC and GCN-RT) and susceptible (GCN-SC and GCN-ST) varieties. This might be due to the fewer data points (3 time-points) from the potato experiment^29^, compared to 8 time-points from the cassava study^25^. Time-series data with over 8 data points offer more precise and highly accurate gene association networks and topological analysis^55, 56^. While the transcriptome data of potato infected with PVY may not be an ideal dataset for the validation, in terms of data resolution, it is one of the very rare time-course data on the response of crops to viral infection in the literature and one that is closely related to cassava. With lacking of data for further consolidation, the findings were strengthened by varying the parameters and statistical criteria (see Additional Information 2) and via multiple computational analyses (Fig. 2, and Additional Information 3A). Availability of more suitable datasets, i.e. long time-course transcriptome data of cassava cultivars with distinct resistance to viral infection, would benefit a proof-of-concept study. Ultimately, this knowledge will help comparatively contrast susceptibility and resistance of cassava varieties to viral infection.

## Conclusions

Cassava plant breeders are constantly in a race to develop cultivars that are resistant to a broad range of important and emerging plant pathogens. However, it is often the case that released cultivars perform inconsistently in different environments, which could be attributed to the lack of comprehensive understanding of the underlying resistance mechanisms and the importance of cooperative gene regulation. In this study, we showed that the resistance of cassava to infection is linked to the clustered network topology of responsive genes in the regulatory systems. Not only the scale-free and small-world global properties are required, but also the local clustering topology of the responsive gene network enables regulatory systems to retain vital functions and promptly respond to infection, as demonstrated by our findings on CBSV resistance in cassava and PVY resistance in potato. Thus, clustered topology of cooperative genes during viral infection is crucial for the robustness of regulatory systems and, ultimately, tolerance. When transcriptome analysis becomes financially affordable and readily accessible, the topology of gene cooperation would help relatively contrast the robustness of cassava response to infection and subsequently be one of the basic criteria for resistant trait selection.

## Material and methods

### Transcriptome data preprocessing

The GCN was constructed from the time-series transcriptome data of CBSV resistant (Namikonga) and susceptible (Albert) cassava cultivars under mock (control) and infection (treatment) conditions (Accession number: PRJNA360340)^25^. Each time point comprised 3 biological replicates studied at different times: 0 (before inoculation), 6, 24, and 48 h post-inoculation (hpi) and at 5, 8, 45, and 54 days post-inoculation (dpi). RNA sequencing was carried out using the Illumina Hi-Seq platform. Data for each time point were separately pre-processed using the transcriptome data analysis pipeline: i) filtering out of low quality reads (Q-score < 28) using Trimmomatic^57^, ii) mapping of high-quality reads to the cassava reference genome (*Mesculenta esculata* version 6.1) retrieved from the Phytozome database, using STAR aligner^58^ at a mapping rate of more than 75%, iii) counting of unambiguously mapped reads by HTseq^59^, iv) normalization of time-series by the relative log expression method (RLE)^60^ and separation of time-series transcriptome data: resistance under control (RC) and treatment condition (RT), susceptible under control (SC) and treatment condition (ST), for the construction gene co-expression networks. Additional Information 1A shows results of the transcriptome data preprocessing according to the pipeline. After the quality control process, one sample (at 45 dpi) was discarded after the trimming process because of extraordinarily low numbers of qualified reads. Quality reads of individual samples were mapped to the cassava reference genome at a mapping rate of 90 percent or greater.

### Gene co-expression network construction

The GCNs were constructed from the active genes and their co-expression. Active genes were defined based on their fluctuation, standard deviation (SD), across the eight time points^61^. The genes with less than 3 expression time points for each condition were excluded from the analysis. Genes with SDs higher than the 77th–80th percentile (depending on datasets, Additional Information 1B) were further assessed for gene–gene association based on the absolute Pearson correlation coefficient ≥ 0.95 and *p*-value < 0.05^56^, using the Hmisc function^62^ in program R 3.6.1^63^. Finally, four gene co-expression networks were constructed to cover the specific conditions: RC, RT, SC and ST. In addition, parameters for the network construction, such as gene selection and gene co-expression selection, were varied to ensure robust results (Additional Information 2A–D). All calculations were performed with the R programming language.

### Network topology analysis

Network topology analysis was performed to measure properties of the regulatory systems. In graph theory, typically a network is a mathematical object that contains *V* vertices, *E* edges and/or *A* arcs. *Node degree* is the number of edges or linkages of each node. High-degree nodes, hub nodes, are important in a network since they have high relationships with others in the system. The *shortest path length* represents the shortest distance between any pairs of nodes, while the *average path length* is the average of all shortest paths between all possible pairs of nodes. The *network diameter* is the longest of the shortest paths between all pairs of nodes in a network. *Betweenness centrality* quantifies the number of times a node acts as a bridge along the shortest path between two other nodes in a network. It ranges between 0 and 1, which respectively quantifies low and high probabilities of the involvement a gene in gene–gene cooperation. Therefore, nodes with high betweenness centrality act as a linkage between neighbor genes in the network, facilitating gene–gene association. *Local clustering coefficient* is a measure of how neighboring nodes are connected to each other. It is computed for each node in the network, and the average of all local clustering coefficients represents the global clustering coefficient of the network. This parameter ranges from 0 to 1, indicating zero to complete connection between neighboring gene nodes of interest, respectively. All networks in this work were topologically analyzed using NetworkAnalyzer. The frequency of network motifs was computed using NetworkMotifDiscovery, and the network was visualized in Cytoscape3.6^64^. Wilcoxon rank-sum test was used for statistical analysis of the topological parameters at *p*-value < 0.05. The random networks with equal numbers of nodes and edges used for the topological analysis were generated by Randomizer^65^.

### Perturbation analysis

In silico perturbation was performed using the perturbR function in program R^66^. Multiple edges were removed sequentially and stochastically. The edges were removed each time at $\alpha =0.02$, where $\alpha$ is proportional edge removal, with $\alpha =0$ denoting no removal and $\alpha =1$ denoting complete removal of all edges. The network perturbation impact was assessed based on the variance information (VI), which is the variation of gene community information (directly and indirectly connected genes) before and after the perturbation, as defined by Karrer and team^66^. The variance information was calculated using: $VI\left(C,{C}^{^{\prime}}\right)=H\left(X|Y\right)+H(Y|X)$, where $VI\left(C,{C}^{^{\prime}}\right)$ is the variance information between gene community before $\left(C\right)$ and after $\left(C^{\prime}\right)$ perturbation, $H\left(X|Y\right)$ is the conditional entropy of $X$ and $Y$, a set of random variables (nodes in GCN; ${x}_{i}$ belongs to node in $C$ and ${y}_{i}$ belongs to node in $C^{\prime}$). The sequential perturbation was performed 30 times, and the mean VI at a particular $\alpha$ was computed to represent the network state at a particular level of perturbation.

### Modular gene network reconstruction

Molecular COmplex DEtection or MCODE is a tool for finding modular clusters in molecular networks using deterministic topological algorithms^67^. The MCODE algorithm begins first calculates a node score based on the connection density of its neighborhood. The highest scored node is assigned as a seed node to initialize the search for potential components in the same module. Then, the algorithm calculates a module score. MCODE iteratively incorporates the possible nodes of the module and recalculates the module score until the score is below the set threshold. In this work, network modules were reconstructed using MCODE with a node score cutoff of 0.6. The modules were iteratively optimized while ensuring that the number of edges within the module with a top MCODE score is more than number of edges between modules. Genes that were not included in the modules were retained as the gene nodes (G-nodes) in the module networks and were linked up with the module nodes (M-nodes). Moreover, we used a robust resolution cutoff, MCODE threshold of 0.5–0.7, for modularization by MCODE (Additional Information 4B–D).

### Functional annotation and analysis of modular gene networks

The gene ontology (GO) annotations were retrieved from the PLAZA database^68^. GO enrichment analysis was performed using GOATOOL^69^, with Bonferroni correction at Q-value < 0.05. The Q-values were transformed to –log (Q-value) and zero for non-enriched functions. The hierarchical clustering was performed using the Weighted Pair Group Method with Arithmetic Mean algorithm (WPGMA) in GENESIS^70^.

### Transcriptional regulatory network (TRN) inference

Transcriptional regulatory networks were inferred from transcription factors (TFs) and their first neighbors in each GCN module. The cassava TFs were annotated from PlantTFDB version 4^71^ and PlantPAN version 2^72^.

### HSP system construction

Heat shock proteins were retrieved from the Pfam protein domain annotations^73^ available in the PLAZA database^68^. They comprised the small HSP family (Pfam id = PF00011), HSP60 family (Pfam id = PF00118), HSP70 family (Pfam id = PF00012), HSP90 family (Pfam id = PF00183), and HSP100 family (Pfam id = PF02861, PF10431 and PF0004). The HSPs and their first-neighbor genes were used to reconstruct the subnetwork of HSPs and their cooperative genes (GCN_HSP-CG_).

### Gene co-expression networks of potato under control and PVY infection conditions

Transcriptome data of potato leaves were retrieved from Goyer et al. (Accession numbers: SRP058212 and SRP058230)^29^. Leaf samples of Premier Russet and Russet Burbank potato cultivars, which are respectively resistant and susceptible to PVY, were collected from control and PVY-infected crops at times 0 (before inoculation), 4 and 10 h post-inoculation (hpi). The transcriptome data were preprocessed by the standard pipeline (see Material and Methods:Transcriptome data preprocessing) and used for constructing the GCNs. Four GCNs were constructed to represent the resistant and susceptible potato under control and treatment conditions, based on the gene fluctuations (SD of gene expression across the 3 time-points, more than 80^th^ percentile of all expressed genes), and their cooperation was inferred by |PCC| with a confidence interval of ≥ 0.95 and *p*-value < 0.05. Predominant genes in the GCNs, denoted by high degree nodes (≥ 500) and high clustering coefficients (c ≥ 0.75), were functionally analyzed using Phytozome^74^ and GOATOOLS^69^.

## Supplementary Information

Supplementary Information.
