# Supplementary material for: Topological clustering of regulatory genes confers pathogenic tolerance to cassava brown streak virus (CBSV) in cassava
Source: Sci Rep. 2021 Apr 12;11:7872. doi: 10.1038/s41598-021-86806-x (PMC8041763; doi:10.1038/s41598-021-86806-x)
Supplement: Supplementary file 1 — Supplementary Information. [file 41598_2021_86806_MOESM1_ESM.pdf]

Topological Clustering of Regulatory Genes Confers Pathogenic Tolerance to Cassava Brown Streak Virus (CBSV) in Cassava

Thanakorn Jaemthaworn[1], Saowalak Kalapanulak[1,2,*], Treenut Saithong[1,2,*]

[1]Bioinformatics and Systems Biology program, School of Bioresources and Technology, School of Information Technology, King Mongkut's University of Technology Thonburi, Bangkok 10150, Thailand
[2]Center for Agricultural Systems Biology, Systems Biology and Bioinformatics Research Group, Pilot Plant Development and Training Institute, King Mongkut's University of Technology Thonburi, Bangkok 10150, Thailand
*treenut.sai@kmutt.ac.th
*saowalak.kal@kmutt.ac.th

Additional Information

Additional Information 1: Transcriptome data processing and GCN network topology analysis

Process	after trimming		Mapped to reference Genome	after trimming		Mapped to reference Genome	after trimming		Mapped to reference Genome	after trimming		Mapped to reference Genome
Name	length	%remained	Uniquely mapped reads %	length	%remained	Uniquely mapped reads %	length	%remained	Uniquely mapped reads %	length	%remained	Uniquely mapped reads %
Condition	SC			ST			RC			RT		
0 h	50	99.51	93.02				50	99.35	93.01			
	50	99.01	92.40				50	98.16	92.82			
	50	99.29	92.64				50	99.81	92.28			
6 hpi*	50	99.81	91.43	50	95.83	91.10	50	99.55	91.19	36-101	99.55	95.69
	50	99.42	90.30	50	98.94	91.24	50	99.13	90.34	36-101	99.80	94.66
	50	99.52	89.37	50	99.79	88.89	50	99.67	91.51	36-101	99.32	95.60
24 hpi	36-101	99.52	95.94	36-101	99.39	93.37	36-101	99.22	95.08	36-101	99.81	94.97
	36-101	99.50	95.86	36-101	99.68	95.28	36-101	99.77	80.58	36-101	99.88	94.81
	36-101	99.26	94.36	36-101	87.50	95.29	36-101	99.35	95.52	36-101	99.84	94.84
48 hpi	36-101	98.27	95.27	36-101	99.51	95.54	36-101	99.00	95.55	36-101	99.36	95.46
	36-101	99.31	96.40	36-101	99.17	95.66	36-101	99.09	92.03	36-101	99.61	95.37
	36-101	99.64	95.57	36-101	99.64	95.77	36-101	99.34	95.34	36-101	99.14	95.68
5 dpi*	36-101	99.87	75.49	36-101	99.69	95.70	36-101	99.36	94.36	36-101	99.77	94.68
	36-101	99.83	95.84	36-101	99.78	79.96	36-101	99.85	94.44	36-101	99.78	94.41
	36-101	99.86	95.84	36-101	91.52	78.98	36-101	99.79	88.13	36-101	99.87	94.83
8 dpi	50	99.62	93.17	50	99.52	92.94	50	99.45	92.05	36-101	99.91	95.33
	50	97.81	91.65	50	99.72	92.81	50	97.13	91.53	36-101	99.89	95.80
	50	98.68	92.58	50	99.83	89.31	50	98.05	92.25	36-101	99.91	95.66
45 dpi	50	11.93	38.18	50	98.73	92.15	36-101	99.79	95.16	50	99.01	91.51
	50	99.05	92.36	50	99.29	88.75	36-101	99.29	94.93	50	98.88	90.53
	50	98.95	92.62	50	99.01	91.90	36-101	99.89	94.68	50	99.86	91.64
54 dpi	50	98.83	91.77	50	97.46	87.26	36-101	99.67	92.69	50	99.71	91.16
	50	98.99	92.47	50	99.43	91.97	36-101	99.67	94.98	50	97.91	91.98
	50	98.68	91.39	50	99.39	91.45	36-101	99.87	95.18	50	99.58	91.13

*hpi: hours post-inoculation
dpi: days post-inoculation

Additional Information 1A Transcriptomics data pre-processing

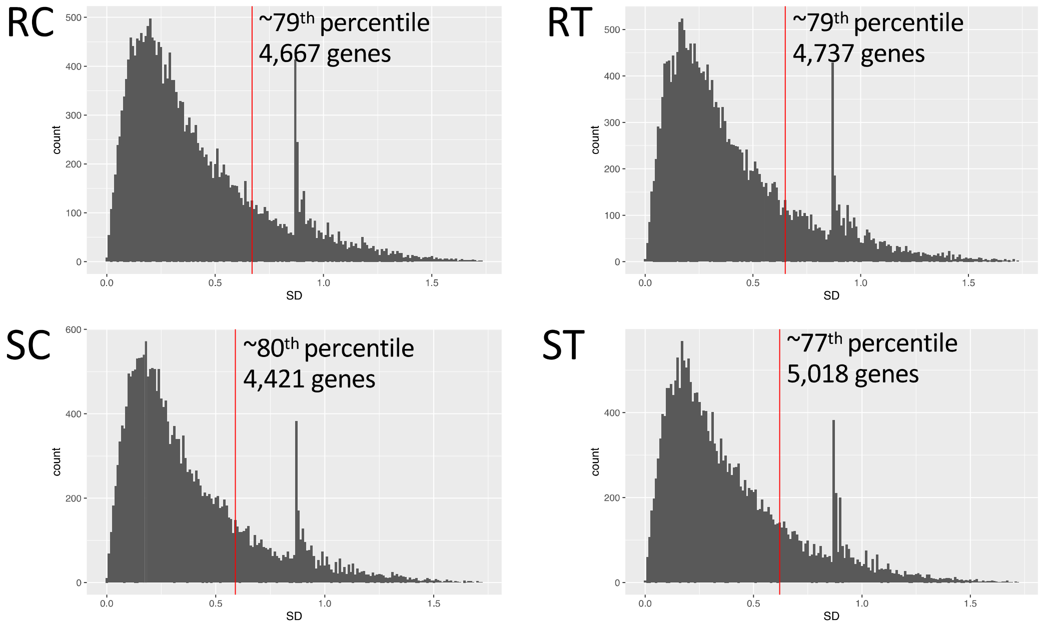

Additional Information 1B Distribution of standard deviation (SD) of gene expression across eight time points of susceptible and resistant cultivars of cassava under control and treatment conditions: susceptible-control (SC), susceptible-treatment (ST), resistance-control (RC), resistance-treatment (RT). The nth percentile and number of genes in each graph determined the statistical cutoff for proposing significant genes in each dataset.

Network properties	GCN				Random			
	GCN-SC	GCN-ST	GCN-RC	GCN-RT	Random-SC	Random-ST	Random-RC	Random-RT
Dimension of network								
· Numbers of nodes (n)	4,142	4,855	4,408	4,418	4,142	4,855	4,408	4,418
· Numbers of edges (e)	124,373	204,227	143,924	87,205	124,373	204,227	143,924	87,205
· Network diameter (d)	28	24	23	26	3	3	3	4
Scale-free properties								
· Power-law distribution ($P(k) \sim k^{-\gamma}$)*	$Y= 996.24x^{-1.070}$ $R^2 = 0.808$	$Y= 791.47x^{-0.964}$ $R^2 = 0.778$	$Y= 847.02x^{-1.047}$ $R^2 = 0.730$	$Y= 1723.1x^{-1.201}$ $R^2 = 0.800$	$Y= 63.854x^{-0.234}$ $R^2 = 0.001$	$Y= 5470.1x^{-1.138}$ $R^2 = 0.022$	$Y= 1.225x^{-0.019}$ $R^2 = 0.015$	$Y= 136.36x^{-0.358}$ $R^2 = 0.004$
Small world properties								
· Average path length (L)	6.413	5.824	7.6	7.272	2.398	2.211	2.36	2.687
· Global clustering coefficient	0.517	0.532	0.539	0.52	0.014	0.017	0.015	0.009

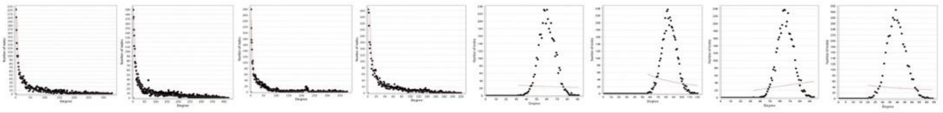

* $P(k) \sim k^{-\gamma}$ is power-law when $P(k)$ is degree distribution of node in the network having k linkages, γ representing constant exponential parameter.

Additional Information 1C Global network topology properties of the condition-specific GCNs compared with random networks detailing the network dimension, scale-free, and small-world properties. All GCNs exhibited scale-free and small-world topological properties. The resistance variety responded to perturbation by decreasing gene associations and increasing the network diameter, while the susceptible one responded by increasing gene associations and decreasing the network diameter.

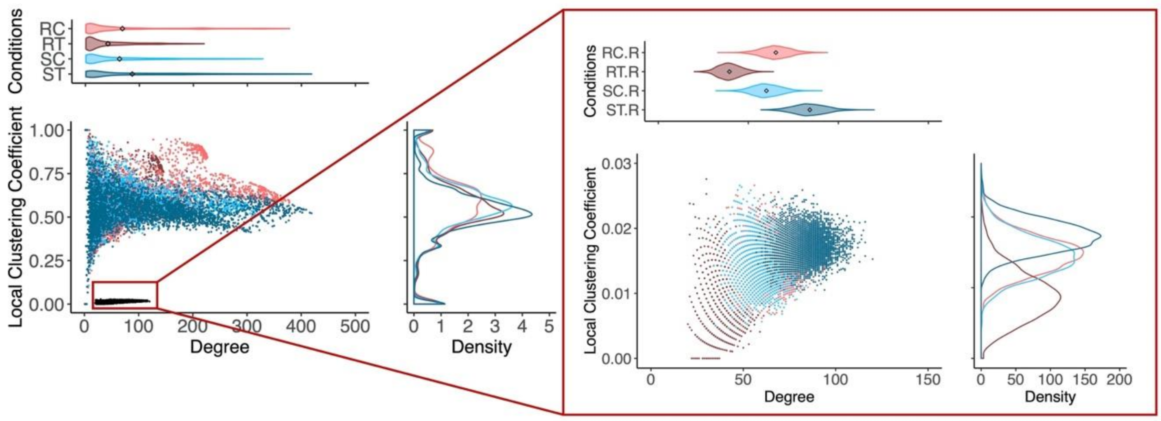

Additional Information1D Local clustering properties of random networks. Distribution of the node degree and local clustering coefficient of the four GCNs and their random networks. The x-axis represents the node degree, and the y-axis represents the local clustering coefficient. The random networks (red box) were vastly different from the GCNs both in terms of magnitude and distribution. Node degree distribution of random networks of both traits responded to the infection similarly as the GCNs, increasing in the susceptible variety and decreasing in the resistant variety, but their local clustering coefficient distribution was noticeably different.

Additional Information 2: GCN network topology of varied parametric criteria

We evaluated the influence of parameters and analytic criteria on the network topology to ensure the robustness and generalization of our findings by varying the gene selection parameters (see results of the topological analysis in Additional Information 2A-B) and gene co-expression selection parameters (see results of the topological analysis in Additional Information 2C-D).

For the gene selection criteria for the GCN construction, we varied the cutoff parameter for the SD: SD>70, SD>80 and SD>90 percentile (P_{70}, P_{80}, P_{90}). The topological analysis of resulting GCNs was subsequently performed as presented previously (Additional Information 1C-D). The networks varied in composition (number of nodes and edges) depending on the cutoffs, but the main inference of the microscopic network topology of the susceptible and tolerant varieties were mostly retained.

For the genes co-expression selection criteria, we varied the confidence interval: $|PCC| > 0.90$, $|PCC| > 0.95$ and $|PCC| > 0.99$, for the analysis of gene sets selected based on the SD > 80 percentile cutoff. The results showed that these criteria had a dominant effect on the number of edges rather than the nodes, especially at |PCC| more than 0.99 (Additional Information 2C-D). Irrespective of the confidence interval, however, the resistant variety showed higher local topological clustering than the susceptible one under control conditions (local clustering coefficient of RC > SC).

(A)

Network properties	Cutoff gene filtering SD > P_{70}, correlation filtering |PCC| > 0.95			
	GCN-SC	GCN-ST	GCN-RC	GCN-RT
Dimension of network				
· Numbers of nodes (n)	6,107	6,266	6,201	6,181
· Numbers of edges (e)	227,778	323,538	209,981	132,200
· Network diameter (d)	23	25	26	29
Scale-free properties				
· Power-law distribution ($P(k) \sim k^{-\gamma}$)*	$Y = 1632.7x^{-1.102}$ $R^2 = 0.829$	$Y = 1202.5x^{-1.010}$ $R^2 = 0.801$	$Y = 1473.5x^{-1.109}$ $R^2 = 0.775$	$Y = 2361.0x^{-1.194}$ $R^2 = 0.837$
	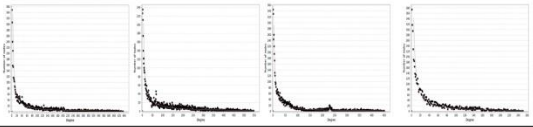			
Small world properties				
· Average path length (L)	5.937	5.575	6.988	6.810
· Global clustering coefficient	0.492	0.494	0.498	0.478

(B)

Network properties	Cutoff gene filtering SD > P_{80}, correlation filtering |PCC| > 0.95			
	GCN-SC	GCN-ST	GCN-RC	GCN-RT
Dimension of network				
· Numbers of nodes (n)	4,056	4,174	4,131	4,115
· Numbers of edges (e)	119,626	155,652	134,007	80,063
· Network diameter (d)	28	24	23	26
Scale-free properties				
· Power-law distribution ($P(k) \sim k^{-\gamma}$)*	$Y = 964.57x^{-1.065}$ $R^2 = 0.805$	$Y = 600.07x^{-0.928}$ $R^2 = 0.743$	$Y = 727.04x^{-1.024}$ $R^2 = 0.725$	$Y = 1211.0x^{-1.116}$ $R^2 = 0.837$
	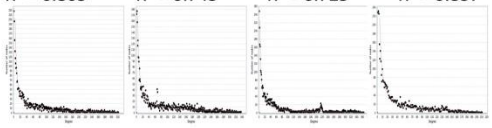			
Small world properties				
· Average path length (L)	6.452	5.960	7.717	7.354
· Global clustering coefficient	0.534	0.524	0.548	0.529

(C)

Network properties	Cutoff gene filtering SD > P_{90}, correlation filtering |PCC| > 0.95			
	GCN-SC	GCN-ST	GCN-RC	GCN-RT
Dimension of network				
· Numbers of nodes (n)	2,044	2,066	2,087	2,057
· Numbers of edges (e)	44,152	46,516	60,069	34,818
· Network diameter (d)	27	24	33	33
Scale-free properties				
· Power-law distribution ($P(k) \sim k^{-\gamma}$)*	$Y = 322.07x^{-0.914}$ $R^2 = 0.746$	$Y = 164.74x^{-0.698}$ $R^2 = 0.531$	$Y = 236.21x^{-0.881}$ $R^2 = 0.605$	$Y = 448.39x^{-1.017}$ $R^2 = 0.775$
	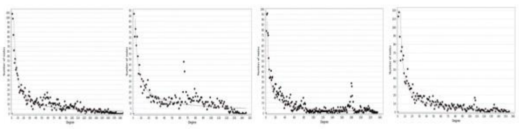			
Small world properties				
· Average path length (L)	7.894	6.685	10.207	10.664
· Global clustering coefficient	0.602	0.582	0.633	0.601

* $P(k) \sim k^{-\gamma}$ is power-law when $P(k)$ is degree distribution of node in the network having k linkages, γ representing constant exponential parameter.

Additional Information 2A Topological analysis of GCNs at different SD cutoffs: (A) SD > 70 percentile, (B) SD > 80 percentile and (C) SD > 90 percentile under |PCC| > 0.95.

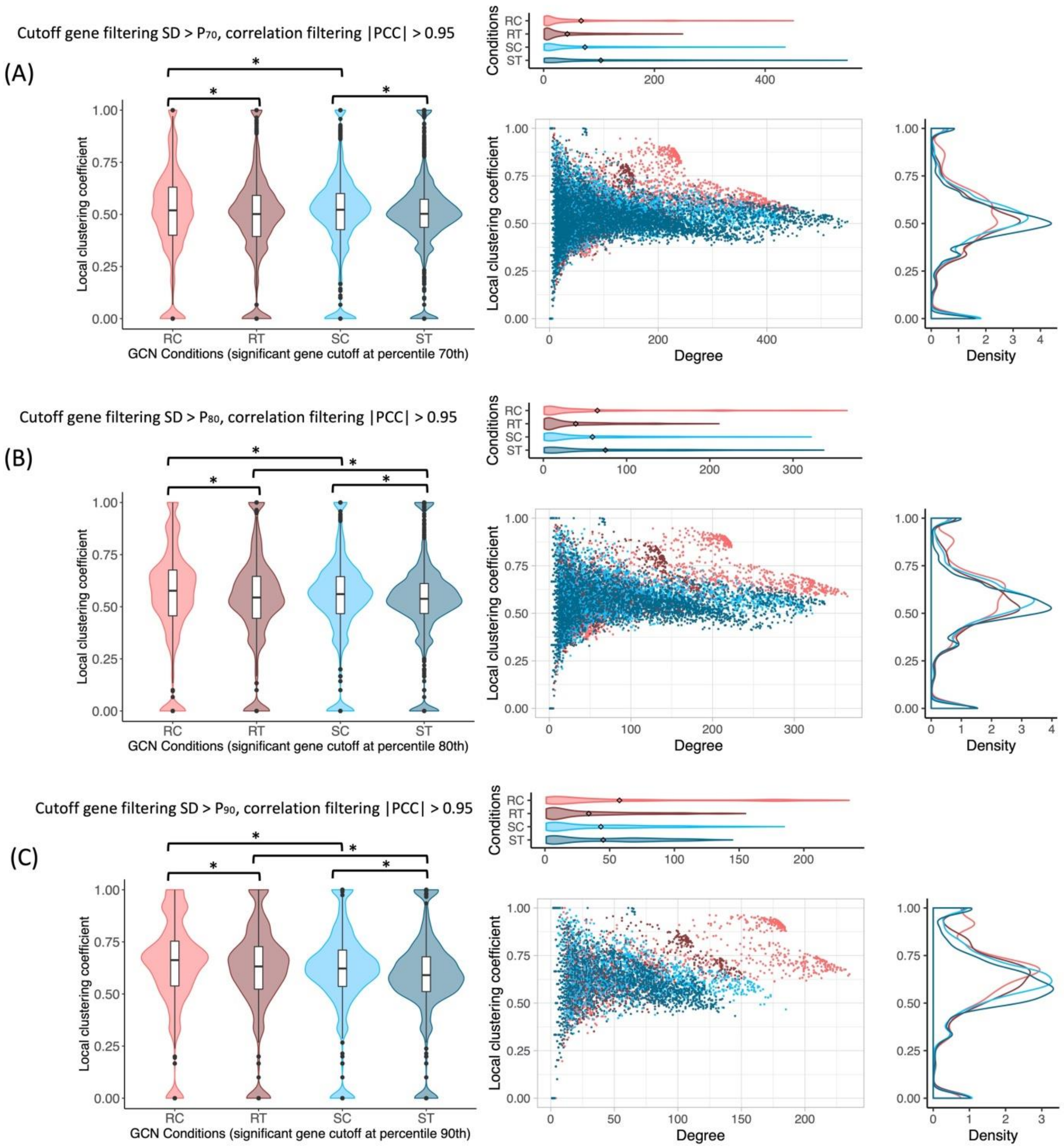

Additional Information 2B Local network properties of the GCNs at different SD cutoffs: (A) SD > 70 percentile, (B) SD > 80 percentile and (C) SD > 90 percentile under |PCC| > 0.95.

(A)

Network properties	Cutoff gene filtering SD > P_{80}, correlation filtering |PCC| > 0.90			
	GCN-SC	GCN-ST	GCN-RC	GCN-RT
Dimension of network				
· Numbers of nodes (n)	4,312	4,302	4,365	4,392
· Numbers of edges (e)	328,276	452,607	312,560	225,012
· Network diameter (d)	14	14	16	17
Scale-free properties				
· Power-law distribution ($P(k) \sim k^{-\gamma}$)*	$Y = 329.65x^{-0.793}$ $R^2 = 0.682$	$Y = 157.24x^{-0.655}$ $R^2 = 0.585$	$Y = 422.33x^{-0.850}$ $R^2 = 0.627$	$Y = 381.36x^{-0.790}$ $R^2 = 0.648$
	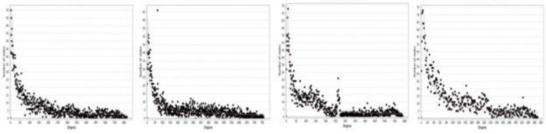			
Small world properties				
· Average path length (L)	4.108	3.926	4.422	4.425
· Global clustering coefficient	0.578	0.580	0.599	0.573

(B)

Network properties	Cutoff gene filtering SD > P_{80}, correlation filtering |PCC| > 0.95			
	GCN-SC	GCN-ST	GCN-RC	GCN-RT
Dimension of network				
· Numbers of nodes (n)	4,056	4,174	4,131	4,115
· Numbers of edges (e)	119,626	155,652	134,007	80,063
· Network diameter (d)	28	24	23	26
Scale-free properties				
· Power-law distribution ($P(k) \sim k^{-\gamma}$)*	$Y = 964.57x^{-1.065}$ $R^2 = 0.805$	$Y = 600.07x^{-0.928}$ $R^2 = 0.743$	$Y = 727.04x^{-1.024}$ $R^2 = 0.725$	$Y = 1211.0x^{-1.116}$ $R^2 = 0.837$
	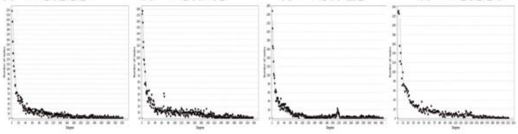			
Small world properties				
· Average path length (L)	6.452	5.960	7.717	7.354
· Global clustering coefficient	0.534	0.524	0.548	0.529

(C)

Network properties	Cutoff gene filtering SD > P_{80}, correlation filtering |PCC| > 0.99			
	GCN-SC	GCN-ST	GCN-RC	GCN-RT
Dimension of network				
· Numbers of nodes (n)	2,385	2,620	2,312	2,157
· Numbers of edges (e)	8,745	10,748	15,767	6,255
· Network diameter (d)	47	31	44	28
Scale-free properties				
· Power-law distribution ($P(k) \sim k^{-\gamma}$)*	$Y = 2077.3x^{-1.682}$ $R^2 = 0.843$	$Y = 1811.6x^{-1.583}$ $R^2 = 0.770$	$Y = 522.89x^{-1.224}$ $R^2 = 0.772$	$Y = 1065.2x^{-1.630}$ $R^2 = 0.858$
	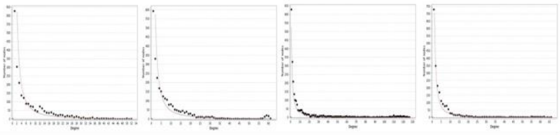			
Small world properties				
· Average path length (L)	11.466	9.874	10.428	7.632
· Global clustering coefficient	0.372	0.386	0.412	0.344

* $P(k) \sim k^{-\gamma}$ is power-law when $P(k)$ is degree distribution of node in the network having k linkages, γ representing constant exponential parameter.

Additional Information 2C Topological analysis of the GCNs at different PCC cutoffs: (A) |PCC| > 0.90, (B) |PCC| > 0.95 and (C) |PCC| > 0.99 under SD of gene expression > 80 percentile.

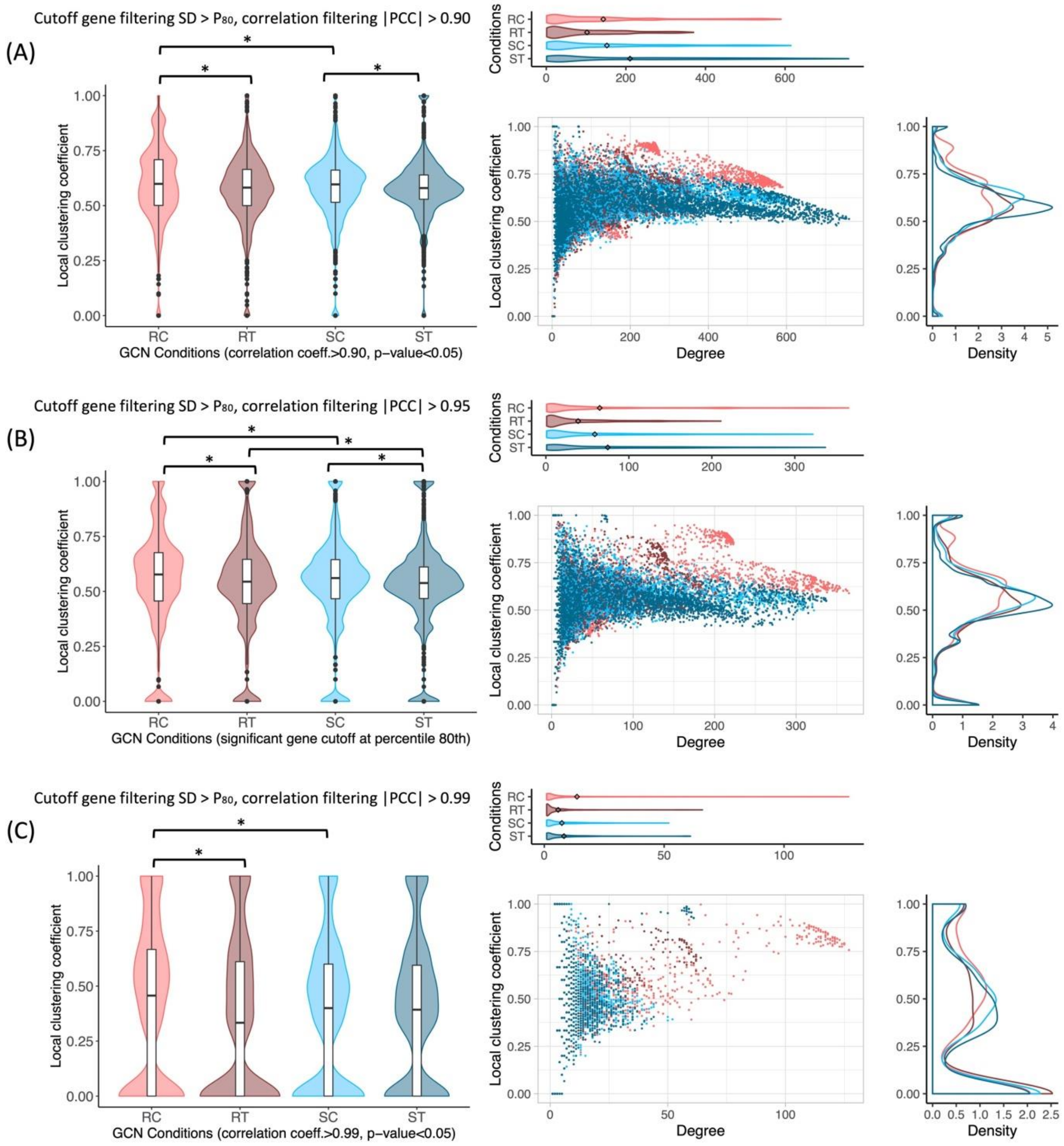

Additional Information 2D Local network properties of the GCNs at different PCC cutoffs: PCC| > 0.90, (B) |PCC| > 0.95 and (C) |PCC| > 0.99 under SD of gene expression > 80 percentile.

Additional Information 3: Network motif discovery analysis

Motif pattern	clustering coefficient	Motif number of real network: RC	Motif number of real network: SC	Motif number of Random RC: $RC_{Random1}$	$RC_{Random2}$	$RC_{Random3}$	Average	Motif number of Random SC: $SC_{Random1}$	$SC_{Random2}$	$SC_{Random3}$	Average
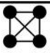	1.00	221,125,826	79,751,428	188	148	174	170.0	95	126	122	114.3
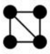	0.83	279,805,505	161,998,749	66,429	68,191	66,725	67,115.0	46,143	46,580	45,918	46,213.7
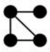	0.58	611,643,046	452,779,373	8,817,761	8,913,851	8,821,974	8,851,195.3	6,301,989	6,324,714	6,276,267	6,300,990.0
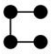	0.00	508,683,630	496,597,639	586,546,972	586,427,961	585,851,625	586,275,519.3	42,944,602	428,884,368	428,826,614	300,218,528.0
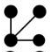	0.00	108,494,577	107,680,629	195,540,003	195,473,866	195,186,943	195,400,270.7	143,220,900	142,936,248	142,894,460	143,017,202.7
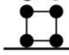	0.00	7,950,219	7,360,665	2,204,720	2,204,994	2,201,342	2,203,685.3	1,582,282	1,577,524	1,579,475	1,579,760.3

Additional Information 3A Network motif discovery analysis of constituent nodes in GCN-RC and GCN-SC. The frequency of network motifs with four nodes in GCN-RC and GCN-SC is presented. The frequency of motifs with high clustering coefficients is higher in GCN-RC than in GCN-SC, and this complex pattern is true specifically for the biological network when compared with the random networks.

Additional Information 4: Analysis of modular gene co-expression networks

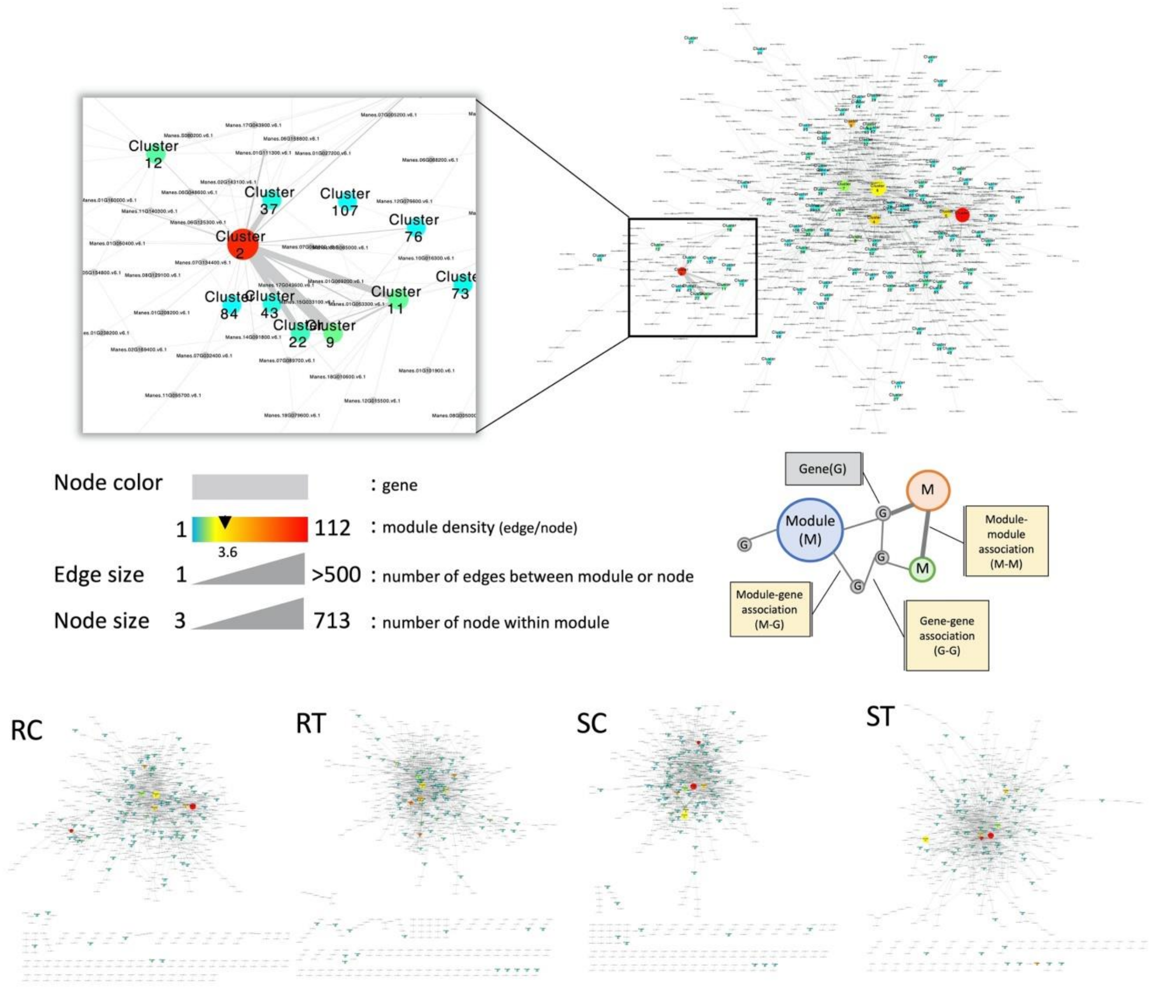

		Module network properties	RC	RT	SC	ST
Module reconstruction	GCN	numbers of nodes	4,408	4,418	4,142	4,855
		numbers of TF nodes	358	326	305	353
		numbers of Edges	143,924	87,250	124,373	204,227
	Module network	number of nodes with assigned modules	3,575	3,558	3,409	4,176
		% node coverage	81.10	80.53	82.30	86.01
		number of TF nodes with assigned modules	283	236	237	295
		% TF node coverage	79.05	72.39	77.70	83.57
		number of edges with assigned modules	116,660	62,857	96,291	140,630
		% edge coverage	81.06	72.04	77.42	68.86
Module network	Number of nodes	all node	936	963	827	760
		module node (M)	111	112	100	85
		funcional enriched module	6	9	7	12
		gene node (G)	825	851	727	675
	Number of edges	all association	1,771	1,835	1,609	1,564
		module-module association (M-M)	199	221	184	155
		module-gene association (M-G)	1,038	1,090	950	946
		gene-gene association (G-G)	534	524	475	463
	Average of	module size (numbers of gene node within module)	32.21	31.77	34.09	49.13
		density (numbers of gene-gene association/numbers of gene node within module)	4.13	3.34	3.38	3.69

Additional Information 4A Modularization of gene co-expression networks and modular gene networks reconstruction. The modules were primarily determined by gene cooperation within groups. Nodes in the modular networks consist of two types, the module node (M) and

gene node (G); the latter represents genes that show no cooperation. The module node size represents the number of genes within the module. The module node color represents density, which is the number of edges divided by the number of nodes within the module. Edges in the modular networks represent inter-module gene cooperation (M-M), cooperation between genes in the module and gene nodes (G-M), and cooperation between the remaining genes (G-G). The edge size represents the number of edges between nodes.

To investigate the effect of parameter settings on the results, parameterization of MCODE for the network modularity was performed because of its role in determining the final topology-based results. The following table (Additional Information 4B and Additional Information 4D) shows the modular networks at varied parameter settings are similar, and more than 90% of genes assigned to the module are within the intersection between each threshold (Additional Information 4C).

		RC			RT			SC			ST		
	MCODE threshold	0.5	0.6	0.7	0.5	0.6	0.7	0.5	0.6	0.7	0.5	0.6	0.7
GCN properties	numbers of nodes	4,408	4,408	4,408	4,418	4,418	4,418	4,142	4,142	4,142	4,855	4,855	4,855
	numbers of Edges	143,924	143,924	143,924	87,250	87,250	87,250	124,373	124,373	124,373	204,227	204,227	204,227
Module properties	number of nodes with assigned modules	3,457	3,575	3,662	3,411	3,558	3,594	3,340	3,409	3,503	4,100	4,176	4,231
	% node coverage	78.43	81.10	83.08	77.21	80.53	81.35	80.64	82.30	84.57	84.45	86.01	87.15
	number of edges with assigned modules	108,429	116,660	125,116	58,253	62,857	70,889	84,684	96,291	105,511	140,519	140,630	163,527
	% edge coverage	75.34	81.06	86.93	66.77	72.04	81.25	68.09	77.42	84.83	68.81	68.86	80.07
	Number of nodes module; module node (M)	119	111	89	150	112	90	119	100	74	94	85	69
	Average of module size (numbers of gene node within module)	29.05	32.21	41.15	22.74	31.77	39.93	28.07	34.09	47.34	43.62	49.13	61.32
	Average of density (numbers of gene-gene association/numbers of gene node within module)	31.37	32.63	34.17	17.08	17.67	19.72	25.35	28.25	30.12	34.27	33.68	38.65

Additional Information 4B Module properties of modular GCNs at different MCODE thresholds

	MCODE Threshold	RC	RT	SC	ST
Numbers of genes with assigned modules	Threshold 0.5	3,457	3,411	3,340	4,100
	Threshold 0.6	3,575	3,558	3,409	4,176
	Threshold 0.7	3,662	3,594	3,503	4,231
Numbers of intersected genes with assigned modules	Intersection	3,343	3,280	3,219	3,958
Percentages of the intersected genes covering genes with assigned modules	Threshold 0.5	96.70	96.16	96.38	96.54
	Threshold 0.6	93.51	92.19	94.43	94.78
	Threshold 0.7	91.29	91.26	91.89	93.55

Additional Information 4C Numbers of intersected genes assigned to the modules at different thresholds

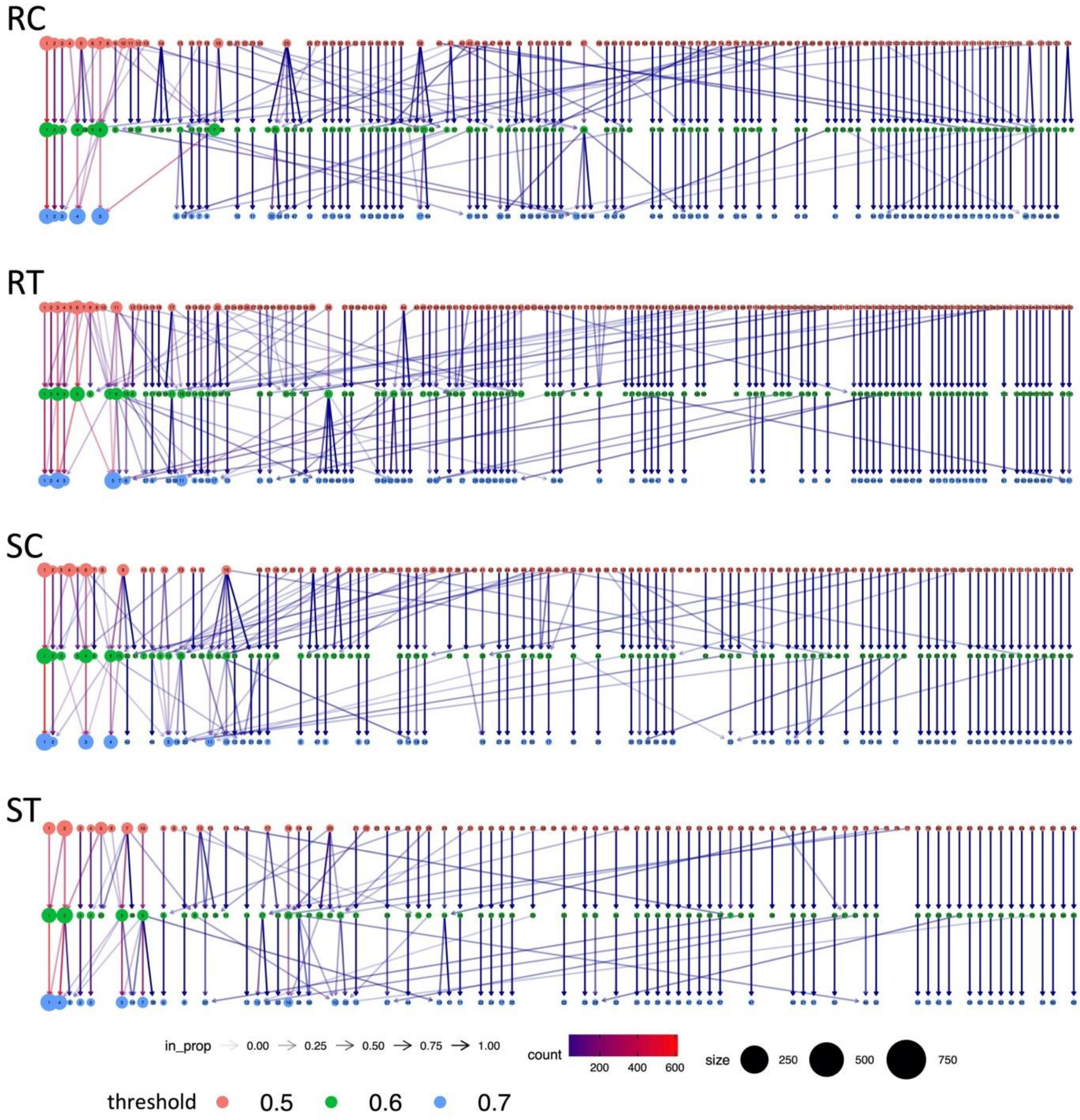

Additional Information 4D Network diagrams of module at different MCODE thresholds. Nodes represent individual modules, node size represents the number of gene nodes in a module, node colors represent the MCODE thresholds, edges represent changes in module genes between the thresholds, edge color represents the number of genes changed by varying the threshold, and edge gradient represents the proportion of genes changed by varying the threshold.

Additional Information 5: Functional analysis of modular gene co-expression networks

A

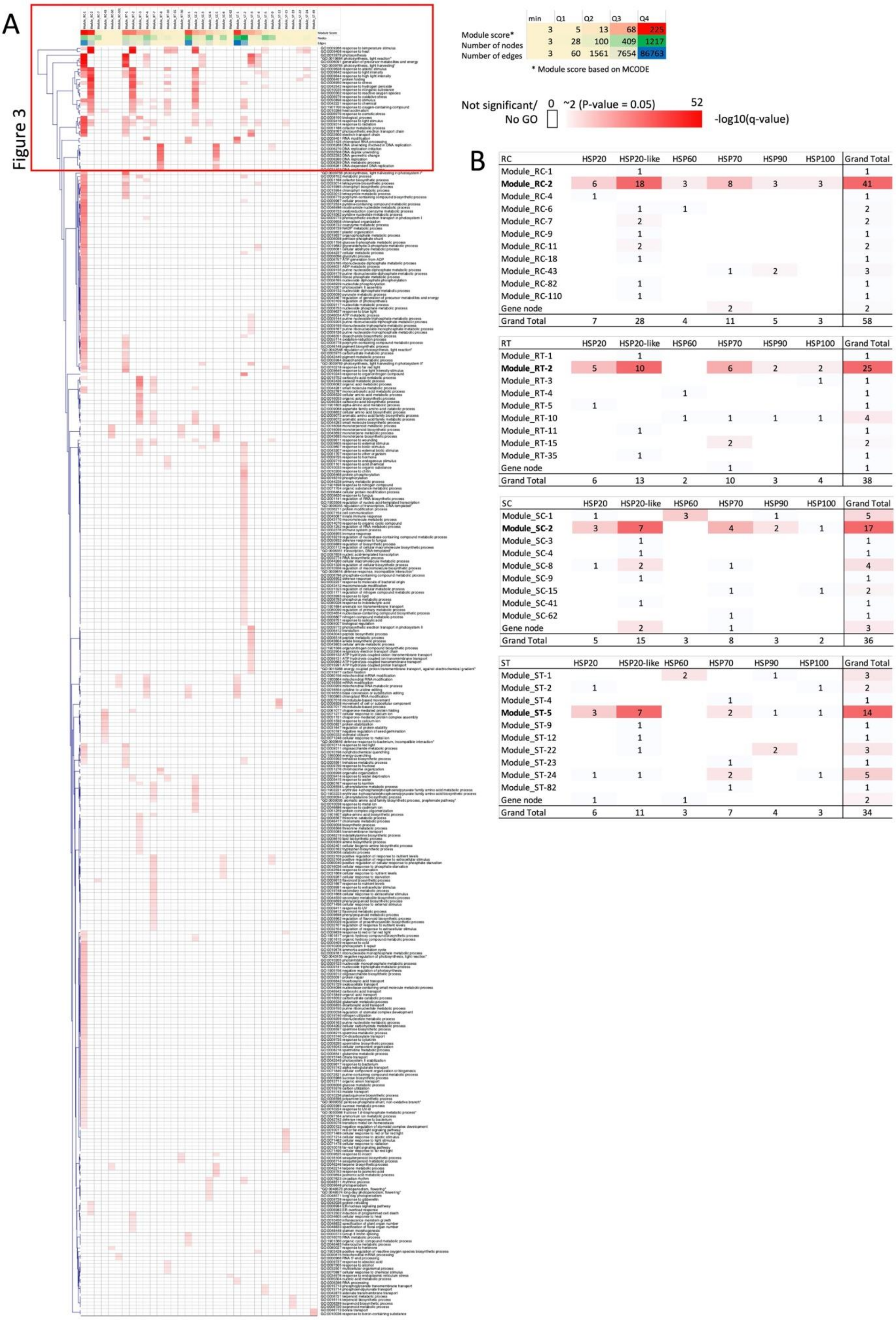

	min	Q1	Q2	Q3	Q4
Module score*	3	5	13	68	225
Number of nodes	3	28	100	409	1217
Number of edges	3	60	1561	7654	86763

* Module score based on MCODE

Not significant/ No GO 0 ~2 (P-value = 0.05) 52 -log10(q-value)

B

RC	HSP20	HSP20-like	HSP60	HSP70	HSP90	HSP100	Grand Total
Module_RC-1		1					1
**Module_RC-2**	6	18	3	8	3	3	41
Module_RC-4	1						1
Module_RC-6		1	1				2
Module_RC-7		2					2
Module_RC-9		1					1
Module_RC-11		2					2
Module_RC-18		1					1
Module_RC-43				1	2		3
Module_RC-82		1					1
Module_RC-110		1					1
Gene node				2			2
Grand Total	7	28	4	11	5	3	58

RT	HSP20	HSP20-like	HSP60	HSP70	HSP90	HSP100	Grand Total
Module_RT-1		1					1
**Module_RT-2**	5	10		6	2	2	25
Module_RT-3						1	1
Module_RT-4			1				1
Module_RT-5	1						1
Module_RT-10			1	1	1	1	4
Module_RT-11		1					1
Module_RT-15				2			2
Module_RT-35		1					1
Gene node				1			1
Grand Total	6	13	2	10	3	4	38

SC	HSP20	HSP20-like	HSP60	HSP70	HSP90	HSP100	Grand Total
Module_SC-1	1		3		1		5
**Module_SC-2**	3	7		4	2	1	17
Module_SC-3		1					1
Module_SC-4		1					1
Module_SC-8	1	2		1			4
Module_SC-9		1					1
Module_SC-15				1		1	2
Module_SC-41		1					1
Module_SC-62				1			1
Gene node		2		1			3
Grand Total	5	15	3	8	3	2	36

ST	HSP20	HSP20-like	HSP60	HSP70	HSP90	HSP100	Grand Total
Module_ST-1			2		1		3
Module_ST-2	1					1	2
Module_ST-4				1			1
**Module_ST-5**	3	7		2	1	1	14
Module_ST-9		1					1
Module_ST-12		1					1
Module_ST-22		1			2		3
Module_ST-23				1			1
Module_ST-24	1	1		2		1	5
Module_ST-82				1			1
Gene node	1		1				2
Grand Total	6	11	3	7	4	3	34

C

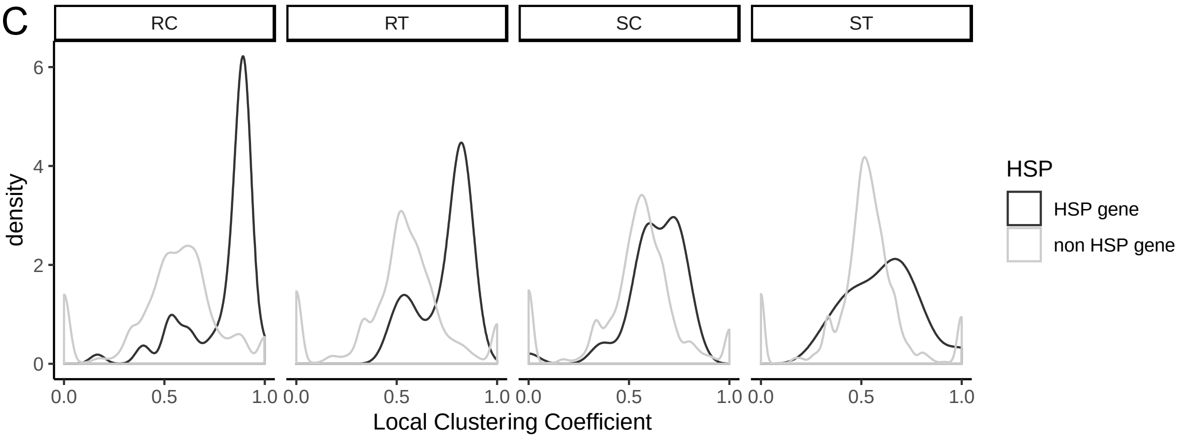

Additional Information 5A Functional analysis of modular gene networks. The GO enrichment analysis of the modular GCNs was conducted based on their constituent genes; (A) Heatmap demonstrating enriched biological process functions of each module. Columns represent modules, rows represent functions (enriched GO IDs), and colors represent the significance (–$\log_{10}$Q-value). Clades with highly significant functions (–$\log_{10}$Q-value more than 10) are highlighted in red boxes (also described in Figure 3). (B) Heatmap demonstrating heat shock protein (HSP) distribution in each module. HSPs, which are related to heat and stress responses in plants, are in the majority in Module_RC-2, Module_RT-2, Module_SC-2, and Module_ST-5. Colors represent the number of HSP genes in the module. (C) Density plot comparing the probabilistic density of local clustering coefficient between HSP genes and non-HSP genes in GCN-RC, GCN-RT, GCN-SC and GCN-ST.

Additional Information 6: Gene expression patterns in functional modules

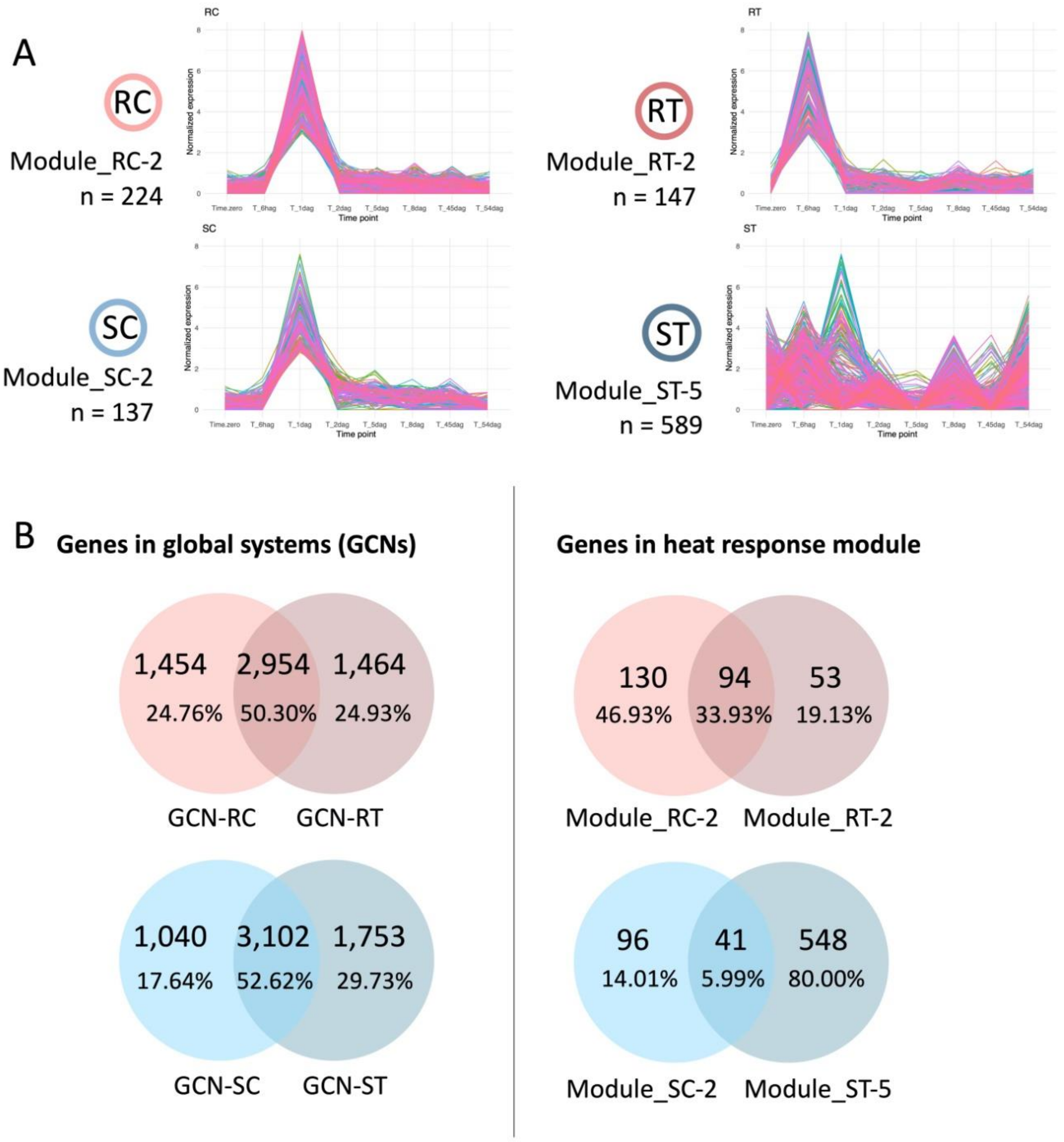

Additional Information 6A Expression patterns of genes in corresponding functional modules (A). Line graph demonstrating the expression time course of genes in the heat response modules (Module_RC-2, Module_RT-2, Module_SC-2, and Module_ST-5). Genes in the control modules responded to the mock graft on day one. For the infected graft, genes in the resistance trait modules (Module_RC-2 and Module_RT-2) rapidly responded at 6 hours after grafting, while genes in the susceptible trait modules (Module_SC-2 and Module_ST-5) showed less cooperation. (B) Venn diagram of genes in the GCNs and heat response modules for each condition. Genes in the resistant network appeared highly conserved after infection, while the susceptible network looked less so.

Additional Information 7: Network motif discovery analysis of functional modules

Motif		Module RC-2	Module SC-2
pattern	clustering coefficient	motif number	motif number
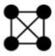	1.00	58,340,445	1,216,791
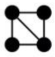	0.83	21,461,696	1,621,703
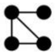	0.58	13,486,121	2,643,909
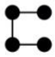	0.00	1,514,361	1,324,572
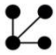	0.00	1,335,260	391,725
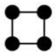	0.00	104,055	59,229
number of nodes		224	137
number of edges		21,812	4,945

Additional Information 7A Network motif discovery analysis of functional modules. Frequency of network motifs in the heat response modules. Based on the frequency of network motifs, Module_RC-2 showed a higher clustering coefficient than Module_SC-2.

Additional Information 8: Association of nucleotide-binding site leucine-rich repeat (NBS-LRR) resistant genes in the modular gene network

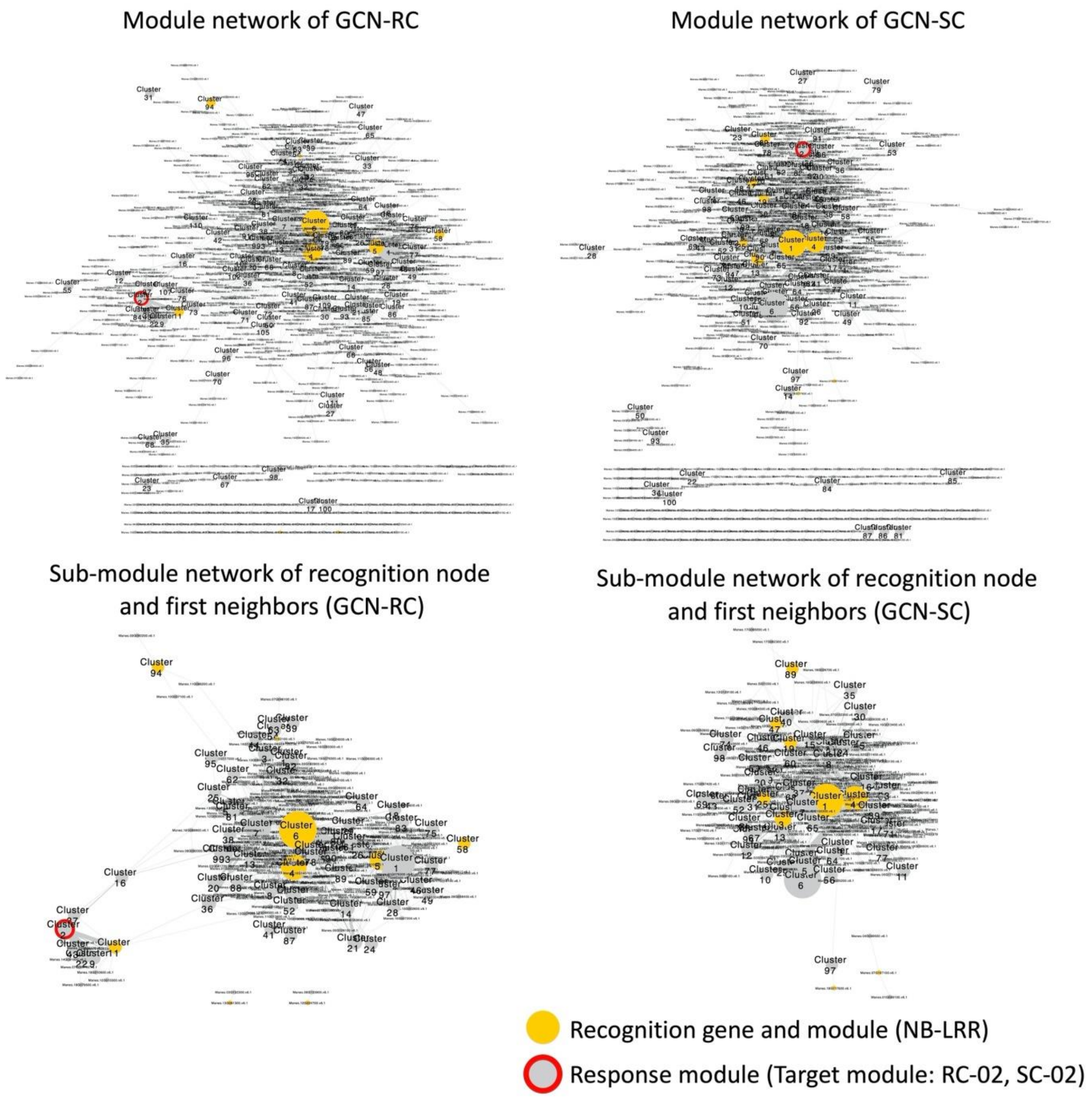

Additional Information 8A Association of nucleotide-binding site leucine-rich repeat (NBS-LRR) resistant genes in the modular gene network of resistant and susceptible cassava cultivars. NBS-LRR resistant genes in the module and gene nodes were annotated and linked to their first neighbors. The subnetworks showed that NBS-LRR genes of resistance cassava could cooperate with the functional response module, while the susceptible cassava could not. This might account for the rapid response of the resistant cassava when infected.

Additional Information 9: **Analysis of TF betweenness centrality in TRN modules**

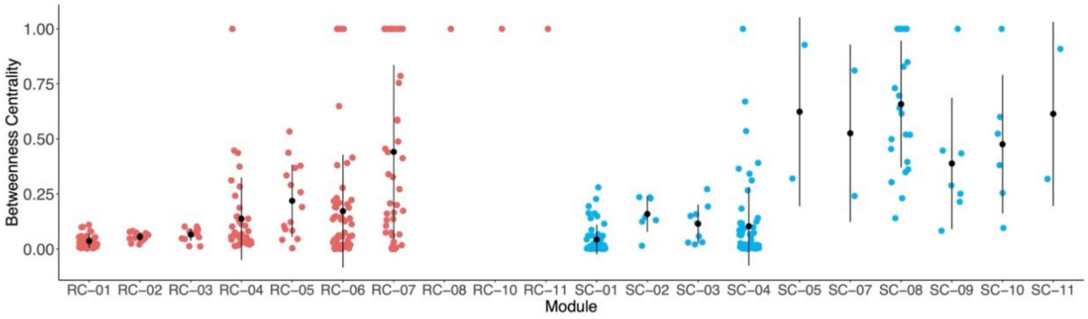

	RC-01	RC-02	RC-03	RC-04	RC-05	RC-06	RC-07	RC-08	RC-10	RC-11
Module Density	112.2	97.4	34.4	21.0	15.7	9.1	4.3	3.9	3.3	3.0
Numbers of TF	33	16	14	37	15	59	41	1	1	1
Average of Degree	206.9	188.9	86.1	49.5	25.4	16.4	8.4	10.0	2.0	2.0
SD of Degree	86.0	27.5	22.0	28.6	18.6	8.3	5.2	NA	NA	NA

	SC-01	SC-02	SC-03	SC-04	SC-05	SC-07	SC-08	SC-09	SC-10	SC-11
Module Density	79.2	36.1	28.5	16.7	10.7	6.5	6.6	6.4	4.2	3.7
Numbers of TF	49	7	9	60	2	2	23	7	6	2
Average of Degree	124.5	70.4	59.7	32.1	18.5	16.5	11.1	11.1	5.3	6.0
SD of Degree	48.3	26.1	17.0	19.5	13.4	6.4	7.9	6.1	3.7	4.2

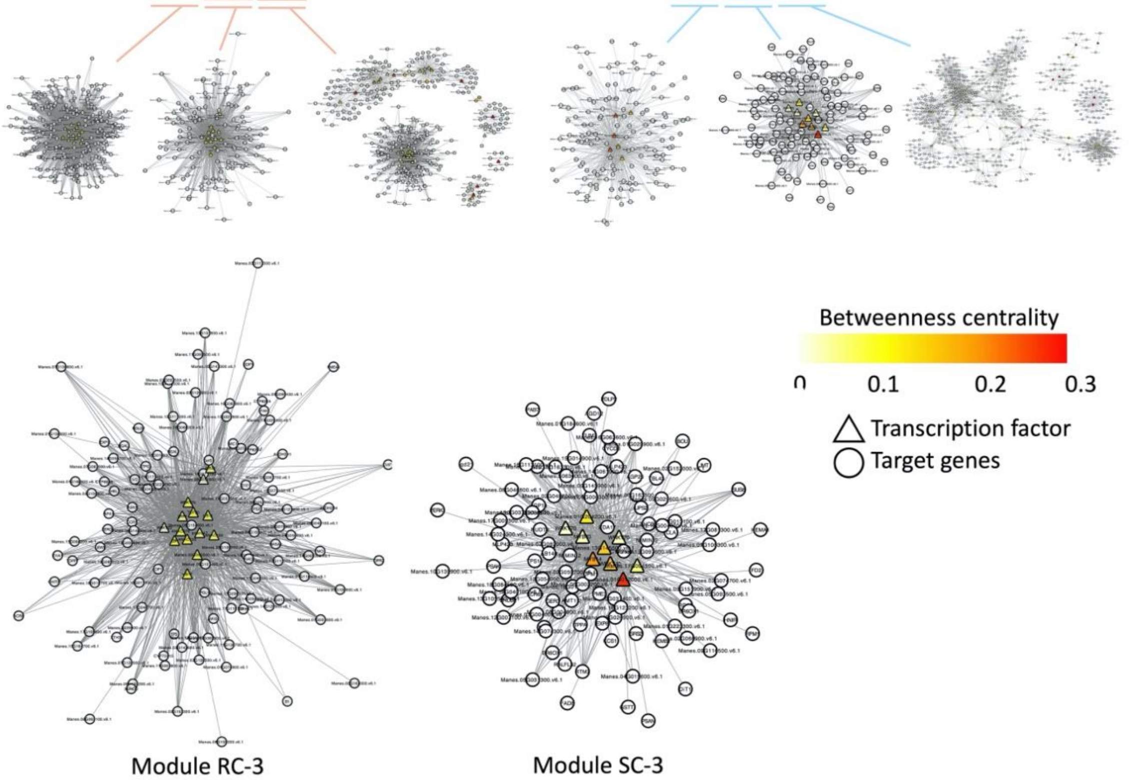

Additional Information 9A Analysis of TF betweenness centrality in TRN modules. Jitter plot of TF betweenness centrality of top ten modules with the highest density, which represents the landscape of TF usage in the modules. TFs with high betweenness centrality were inferred to be highly required in the module. The standard deviation (SD) of TFs' degree indicates of the importance of TFs in the module; low SD denotes a considerable influence of TFs in the module, which generally enables the flexibility and robustness of the regulatory system, while high SD denotes a large variation in the influence of TFs, which implies that the system relies on a few crucial TFs.

Additional Information 10: Gene co-expression networks of HSP systems and their topological analysis.

	Proposed model	Heat shock proteins subnetwork (GCN_{HSP})	Heat shock proteins and target genes subnetwork ($GCN_{HSP\text{-}CG}$)
RC	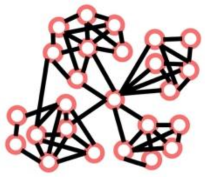	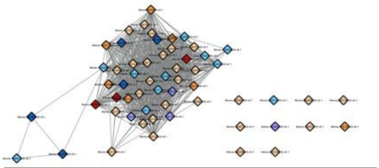<br>Number of nodes 58<br>Number of edges 795<br>Global clustering coefficient 0.77	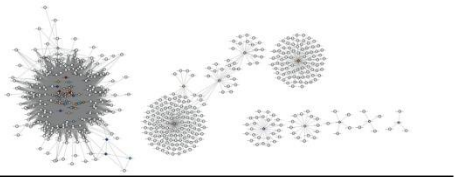<br>Number of nodes 591<br>- HSP nodes 58<br>- Target nodes 533<br>Number of edges 8232<br>Global clustering coefficient 0.36
RT	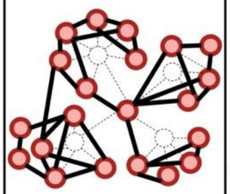	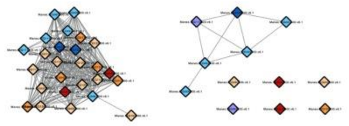<br>Number of nodes 38<br>Number of edges 289<br>Global clustering coefficient 0.74	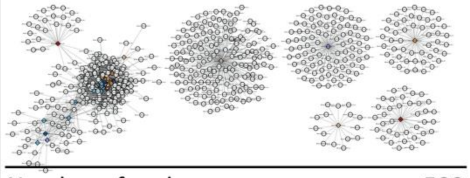<br>Number of nodes 592<br>- HSP nodes 38<br>- Target nodes 554<br>Number of edges 3450<br>Global clustering coefficient 0.25
SC	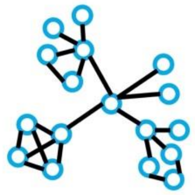	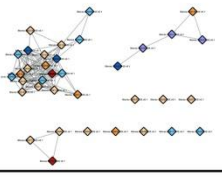<br>Number of nodes 36<br>Number of edges 128<br>Global clustering coefficient 0.59	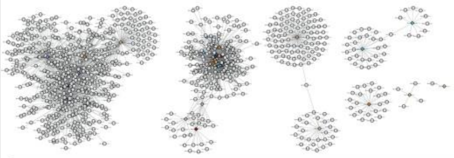<br>Number of nodes 909<br>- HSP nodes 36<br>- Target nodes 873<br>Number of edges 2563<br>Global clustering coefficient 0.38
ST	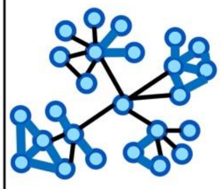	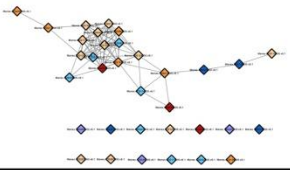<br>Number of nodes 34<br>Number of edges 78<br>Global clustering coefficient 0.4	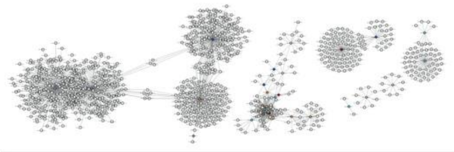<br>Number of nodes 1052<br>- HSP nodes 34<br>- Target nodes 1018<br>Number of edges 1559<br>Global clustering coefficient 0.05

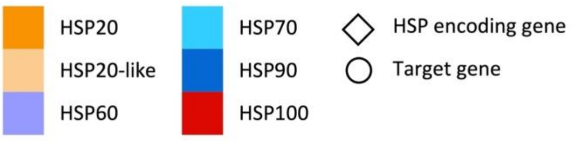

Additional Information 10A Gene co-expression networks of HSP systems and their topological analysis. The HSP subnetwork (GCN_{HSP}) was reconstructed from mapped HSP-encoding genes in the GCNs; the first neighbor genes and targets were incorporated to obtain the HSP and target gene subnetwork ($GCN_{HSP\text{-}CG}$). Topological analysis showed that the global clustering coefficient for the resistant GCN_{HSP} was higher than for the susceptible one in both control and infection conditions

Additional Information 11: Gene co-expression networks and topological properties of Potato Virus Y response in resistant and susceptible potato cultivars

Network properties	GCN				Random			
	GCN-SC	GCN-ST	GCN-RC	GCN-RT	Random-SC	Random-ST	Random-RC	Random-RT
Dimension of network								
· Numbers of nodes (n)	4,495	4,479	4,440	4,437	4,495	4,479	4,440	2,237
· Numbers of edges (e)	1,123,337	1,127,386	1,262,071	1,236,227	1,123,337	1,127,386	1,262,071	1,236,227
· Network diameter	12	12	12	12	2	2	2	2
Small world properties								
· Average path length (L)	5.766	5.775	5.597	5.624	1.889	1.888	1.872	1.874
· Global clustering coefficient	0.761	0.761	0.767	0.768	0.111	0.112	0.128	0.126

Additional Information 11A The global network properties of the GCNs and random networks for the resistant and susceptible potato cultivars under control and PVY conditions.

Our findings on the importance of the network architecture, particularly topological clustering of GCNs, for systems' robustness during perturbation and CBSV resistance in cassava, is corroborated by the study on transcriptional regulatory networks (TRNs) of CBSV resistance in casava[1]. Additionally, the validation was performed by studying the GCNs of potato response to PVY, employing time-series transcriptome data. Additional data processing was applied to minimize any effects of the small number of data points. First, the active genes were determined based upon changes in their expression across 3 time-points, and the top 20 percentile of the highly fluctuating expression (SD > 80th percentile of all expressed genes). The differentially expressed genes were mapped to potato TRNs from the PlantRegMap database[2], and the associated TFs and target genes (TGs) were determined by Pearson's correlation. Then, the TRNs for the individual conditions were inferred (TRN-SC, TRN-ST, TRN-RC, and TRN-RT) from the gene associations with PCC > 0.6 and PCC < -0.9. The criteria were defined according to the distribution of PCC among studied genes (Additional Information 11B). Additional Information 11C shows the characteristics of the resulting TRNs.

Topological analysis of the potato TRNs focused mainly on the association of TFs because those of TGs were incompletely reconstructed as PlantRegMap did not satisfy the assumptions for this analysis. Additional Information 11D and Additional Information 11E show that the local clustering coefficient of RC is significantly greater than for SC, and the local clustering coefficient of RT was significantly greater than for ST (Wilcoxon rank-sum one-sided test, p-value < 0.05). Although differences in the local clustering coefficients for control and treatment conditions were not significant, the control generally showed higher local clustering coefficient and global clustering coefficient distributions (SC > ST and RC > RT). These are consistent with our conclusion that biological robustness is linked to the higher local clustering coefficient observed in control conditions (RC > SC, SC > ST and RC > RT).

---

1. Jaemthaworn, T., Saithong, T. & Kalapanuluk, S. Dissecting the transcriptional regulatory network to reveal beneficial network topology for cassava brown streak virus resistance. in *The 31st Annual Meeting of the Thai Society for Biotechnology and International Conference* 335–347 (2019). doi:https://doi.org/10.14455/tsb.res.2019.21

2. Tian, F., Yang, D., Meng, Y., Jin, J. & Gao, G. PlantRegMap : charting functional regulatory maps in plants. *Nucleic Acids Res.***48**, 1104–1113 (2020).

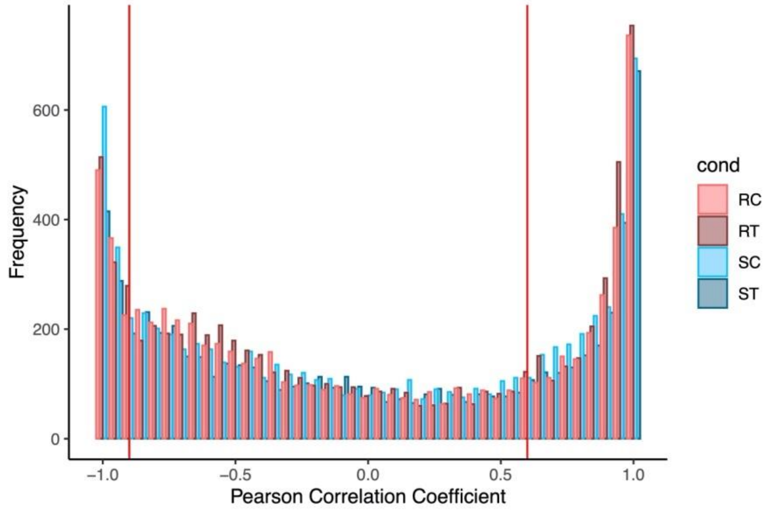

Additional Information 11B Histogram of Pearson correlation coefficients of differentially expressed genes in TRNs of tolerance and susceptible potato varieties.

	Nodes	Edges
TRN-RegNet	33,031	288,000
TRN-SC	1,672	2,901
TRN-ST	1,667	2,489
TRN-RC	1,756	2,764
TRN-RT	1,849	2,959

Additional Information 11C Characteristics of TRNs of tolerance and susceptible potato varieties demonstrating TF-TG associations.

Network properties	TRN$_{TF-TF}$ conditions			
	TRN$_{TF-TF}$-SC	TRN$_{TF-TF}$-ST	TRN$_{TF-TF}$-RC	TRN$_{TF-TF}$-RT
Dimension of network				
· Numbers of nodes (n)	173	163	175	177
· Numbers of edges (e)	255	230	263	293
· Network diameter (d)	9	10	8	9
Scale-free properties				
· Power-law distribution $(P(k) \sim k^{-\gamma})$*	$Y = 74.046x^{-1.340}$ $R^2 = 0.875$	$Y = 68.094x^{-1.425}$ $R^2 = 0.917$	$Y = 62.587x^{-1.314}$ $R^2 = 0.905$	$Y = 61.384x^{-1.379}$ $R^2 = 0.832$
	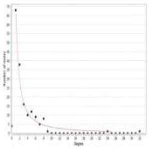	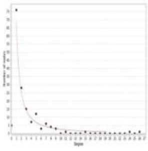	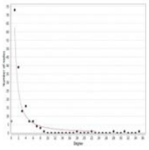	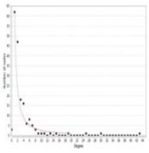
Small world properties				
· Average path length (L)	4.379	4.230	3.647	3.707
· Global clustering coefficient	0.082	0.031	0.114	0.063

Additional Information 11D Topological analysis of TF-TF associations in TRNs of tolerance and susceptible potato varieties.

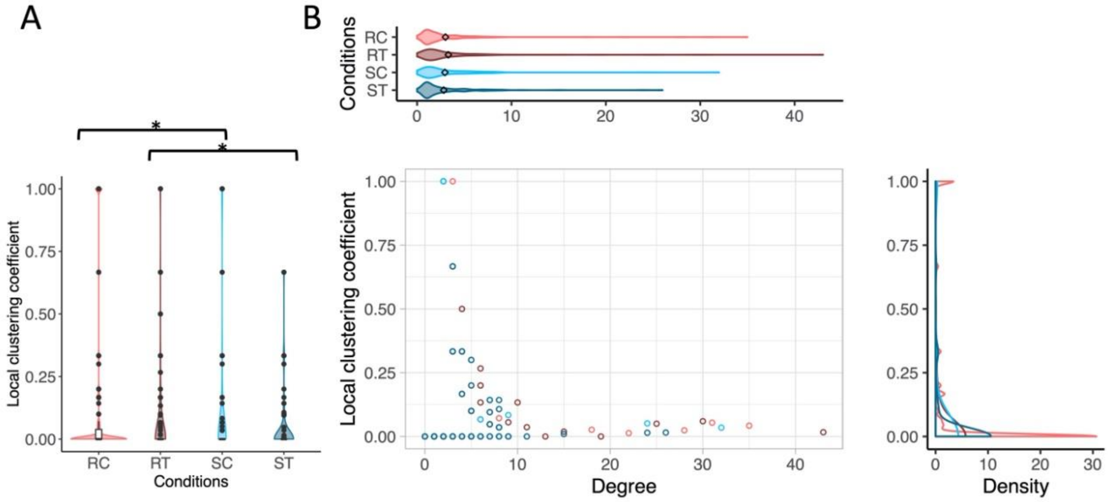

Additional Information 11E Local network properties of TF-TF associations in TRNs of tolerance and susceptible potato varieties. (A) Violin plot demonstrate significantly different of local clustering coefficient distribution (star; *, indicate statistically different by Wilcoxon rank-sum test, p-value < 0.05). (B) Scatter plot demonstrate distribution of local clustering coefficient and degree of nodes in TRN$_{TF-TF}$
